# Hyaluronic acids mediate the infiltration, migration, and M2 polarization of macrophages: evaluating metabolic molecular phenotypes in gliomas

**DOI:** 10.1002/1878-0261.13315

**Published:** 2022-10-10

**Authors:** Hao Zhang, Nan Zhang, Ziyu Dai, Zeyu Wang, Xun Zhang, Xisong Liang, Liyang Zhang, Songshan Feng, Wantao Wu, Weijie Ye, Jian Zhang, Peng Luo, Zaoqu Liu, Quan Cheng, Zhixiong Liu

**Affiliations:** ^1^ Department of Neurosurgery, Xiangya Hospital Central South University Changsha China; ^2^ National Clinical Research Center for Geriatric Disorders, Xiangya Hospital Central South University Changsha China; ^3^ Department of Neurosurgery, The Second Affiliated Hospital Chongqing Medical University China; ^4^ One‐third Lab, College of Bioinformatics Science and Technology Harbin Medical University China; ^5^ Department of Oncology, Xiangya Hospital Central South University Changsha China; ^6^ Department of Clinical Pharmacology, Xiangya Hospital Central South University Changsha China; ^7^ Department of Oncology, Zhujiang Hospital Southern Medical University Guangzhou China; ^8^ Department of Interventional Radiology The First Affiliated Hospital of Zhengzhou University China

**Keywords:** cell communication, gliomas, hyaluronic acid, macrophage, metabolism, tumor microenvironment

## Abstract

Gliomas cause high mortality around the world. The metabolic pattern of the tumor was previously suggested to be associated with the patient's survival outcome and immune activity. Yet, this relationship in glioma remains unknown. This study systematically evaluated the immune landscape in different phenotypes classified by metabolic‐related pathways of 3068 glioma samples and 33 glioblastoma single‐cell sequencing samples. Machine learning prediction analysis of microarray with R (pamr) was used for validating clustering results. A total of 5842 pan‐cancer samples were used for external validation of the metabolic clusters. Cell Counting Kit‐8 (CCK8) assay, cell clone assay, EdU assay, wound healing assay, Transwell assay, and co‐culture assay were performed to verify the distinction in molecular characteristics among metabolic clusters. Metabolomics and RNA sequencing were performed on HS683 and U251 cells to annotate potential hyaluronic acid (HA)‐mediated pathways. Three distinct metabolic phenotypes were identified. Metabolic cluster 1 correlated with a high number of immune infiltrating cells and poor survival of glioma patients. Metabolic clusters were proved with different levels of the macrophage markers CD68 and CD163 by multiplex immunofluorescence staining. Glioma cells from other metabolic clusters also expressed various levels of HA. HA was further found to mediate glioma proliferation, progression, and invasion. Moreover, HA potentially promoted macrophage recruitment and M2 polarization through the IL‐1/CHI3L1 and TGF‐*b*/CHI3L1 axes. HA also regulated the expression of PD‐L1. This work revealed the significant connection between metabolic patterns, especially HA, and tumor immune infiltration in gliomas.

## Graphical abstract

This study developed three metabolic clusters based on metabolic‐related pathways that enabled us to comprehensively explore the interconnection between metabolism and TME cells in individual glioma patients. Metabolic clusters showed differences in HA that significantly promoted the proliferation, migration, invasion of GBM, and infiltration and recruitment of macrophages through the IL‐1/CHI3L1 and TGF‐β/CHI3L1 axis.

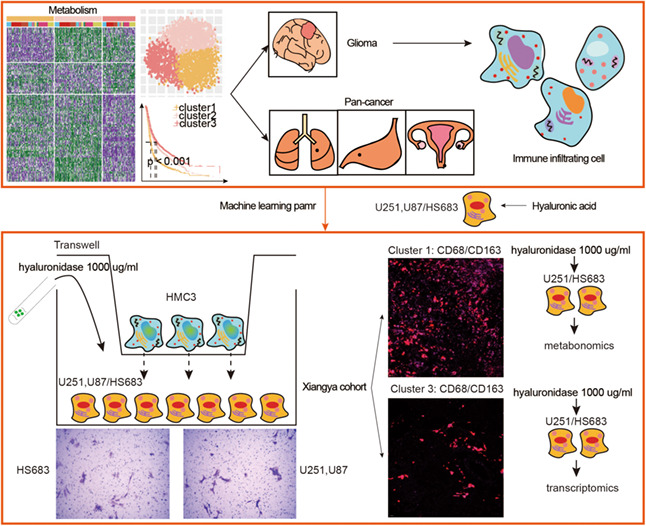

**Abbreviations**
APM: antigen processing and presenting machinery
CCLE: Cancer Cell Line Encyclopedia
CYT: cytotoxic activity
DC: dendritic cells
DEG: differentially expressed genes
DMEM: Dulbecco's modified Eagle's medium
FDR: false discovery rate
FPKM: fragments per kilobase million
GBM: glioblastoma
GEO: Gene Expression Omnibus
GEP: T cell‐inflamed gene expression profile
GO: Gene Ontology
GSEA: gene set enrichment analysis
GSVA: gene set variation analysis
HA: hyaluronic acid
HRD: homologous recombination deficiency
ICB: immune checkpoint blockage
IFNG: interferon gamma
IFNG.GS: IFNG hallmark gene set
ISG.RS: interferon‐stimulated genes resistance signature
LGG: low grade glioma
OS: overall survival
pamr: prediction analysis of microarray with R
PCA: principal component analysis
RMA: robust multichip average
TCGA: The Cancer Genome Atlas
TCR: T cell receptor
TMB: tumor mutation burden
TME: tumor microenvironment
TPM: transcripts per kilobase million
WHO: World Health Organization

## 1 Introduction

Metabolic activities have been proven to be altered in cancer cells relative to normal cells. The reprogrammed metabolism greatly supports maintaining and enhancing malignant properties in multiple cancers [1]. Classical reprogrammed activities, such as altered bioenergetics, enhanced biosynthesis, and redox balance, may support cancer cell survival and proliferation under stressful conditions [2]. The crosstalk between metabolic networks and tumorigenic signaling has provided a new prospect for investigating suitable therapeutic targets. Aerobic glycolysis, also known as the Warburg effect, has received the most attention among the complex metabolic networks. This feature is characterized by hypoxia and mitochondrial abnormalities, which involve proliferating cancer cells' propensity to transform glucose into lactate [3]. Thus, the Warburg effect ensures the rapid and unbridled proliferation rate of tumor cells.

Gliomas represent one of the most lethal solid tumors in the central nervous system. Based on the 2021 World Health Organization (WHO) classification [4], patients with low‐grade glioma (LGG) have a median overall survival (OS) of 8–10 years, and patients with glioblastoma (GBM) only have an overall survival of approximately 12–14 months after surgery with adjuvant chemoradiotherapy [5]. The dismal clinical outcome of GBM makes the research on novel therapeutic targets challenging and urgent [5]. Previous studies have demonstrated that the Warburg effect could facilitate the proliferation of GBM cells, as glucose is the dominant growth substrate in GBM [6]. In addition, GBM cells utilize the tricarboxylic acid cycle/oxidative phosphorylation in a different capacity than normal brain tissue [7], and GBM cells have also been shown to use fatty acids, glutamine, and urea cycle metabolites to meet the high demand for ATP consumption [8]. Notably, targeting glutamine metabolism has been considered a potential therapeutic option in GBM [9]. Thus, the interconnection between metabolism and gliomas suggests the possibility of classifying gliomas from the metabolic perspective and subsequently identifying metabolic phenotypes performing distinct functions.

Previous studies have proved that different immune cells have distinct metabolic requirements. T cells, macrophages, and dendritic cells in the tumor microenvironment (TME) have significantly specific plasticity to engage in different metabolic‐related pathways with altered or impaired immune functions [10, 11]. Subsequently, the altered immune infiltrating cells could create either permissive or hostile TME that affects the survival and proliferation of tumor cells. Previous studies have proved that the immune infiltrating microenvironment regulates the proliferation, migration, and progression of glioma cells [12]. The immunomodulatory mechanisms of gliomas pose a challenge to the rising immunotherapy represented by immune checkpoint blockage (ICB) [13]. Therefore, establishing the connection between metabolism and the immune infiltrating microenvironment of gliomas is significant.

To date, characterization of the metabolic phenotypes of gliomas and their interconnection with the immune infiltrating microenvironment has not been reported. This study developed a metabolism‐related signature in gliomas and investigated the correlation between glioma metabolic activity profile and cancer immunity.

## 2 Materials and methods

### 2.1 Dataset collection and preprocessing

The glioma gene expression profiles and the corresponding clinical datasets were collected from the Gene Expression Omnibus (GEO; http://www.ncbi.nlm.nih.gov/geo/), The Cancer Genome Atlas (TCGA; https://xenabrowser.net/), and Chinese Glioma Genome Atlas (CGGA; http://www.cgga.org.cn/). Pan‐cancer data were from the TCGA dataset, and 5842 pan‐cancer samples were included in this study. A total of 3068 glioma patient samples were collected from 13 cohorts (Tables S1 and S2). The following search terms were used: (((survival OR prognosis OR prognostic OR outcome OR death OR relapse OR recurrence))) AND (Glioma*[Title]) OR (Astrocytoma*[Title]) OR (Glioblastoma*[Title]) OR (Ependymoma*[Title]) OR (Oligodendroglioma*[Title]) OR (Gliosarcoma*[Title]) OR (Astroglioma*[Title]) OR (LGG*[Title]) OR (HGG*[Title]) OR (glial cell tumor*[Title]). All publicly available glioma datasets with the corresponding clinical annotations were manually examined. Patients lacking survival information were excluded from further evaluation.

Raw data from GEO datasets were generated using affymetrix and agilent microarrays. The robust multichip average (RMA) algorithm was used to perform quantile normalization and background correction of the raw data from affymetrix microarray. Regarding affymetrix microarray, the consensus median polish algorithm was used for the final summarizing of oligonucleotides for each transcript. The package ‘LIMMA’ was used for processing the raw data from agilent. Bulk sequencing data were downloaded from the TCGA and CGGA data portals. The fragments per kilobase million (FPKM) values were transformed into transcripts per kilobase million (TPM) values with similar signal intensity to the RMA‐standardized values from GEO datasets. The r package sva was used to remove the computational batch effect among each cohort. Each cohort was processed and normalized independently. The methods have also been described in an earlier paper [14].

Glioma tissues were collected and written informed consent was obtained from all patients. The included glioma tissues were approved by the Ethics Committee of Xiangya Hospital, Central South University. The study methodologies conformed to the standards set by the Declaration of Helsinki.

### 2.2 Unsupervised consensus clustering for metabolic‐related pathways

The 115 metabolism‐relevant gene signatures were achieved from a previously published study [15]. Each sample received the enrichment score from the 115 metabolism‐relevant gene signatures using the gsva r package. Tumors with diverse enrichment patterns of metabolic‐related pathways were classified using the consensus cluster method, which grouped patients with different metabolic patterns for further analysis. The optimal number of clusters and their stability and reliability in the meta‐cohort and TCGA cohort were determined using the consensuclusterplus r package.

### 2.3 Estimation of immune infiltrating cells

The xCell algorithm [16] was used to quantify the abundance of immune cells in glioma samples. It allowed for sensitive and specific discrimination of 40 human infiltrating immune cell phenotypes, including T and B cells, dendritic cells, macrophages, natural killer cells, and myeloid cell subtypes. The timer algorithm [17], epic algorithm [18], mcpcounter algorithm [19], quantlseq algorithm [20], and cibersort algorithm [21] were also used for identifying immune infiltrating cells.

### 2.4 Pathway enrichment analysis

All gene sets were downloaded from the MSigDB database [22]. Gene set variation analysis (GSVA) was performed on the metabolic clusters in meta‐cohort and TCGA using the gsva r package [23]. Pathways enriched in metabolic clusters were identified in Gene Ontology (GO) with the false discovery rate (FDR) < 0.05 and a strict cutoff of *P* < 0.01. Gene set enrichment analysis (GSEA) was performed on the metabolic clusters in single‐cell sequencing datasets using the clusterprofiler r package [24].

### 2.5 Estimation of immune modulators

Seven types of immune checkpoints were collected from previously published work [25]. T cell‐inflamed gene expression profile (GEP) was defined based on the expression of the 18 genes [26]. Cytotoxic activity (CYT) was determined based on the gene expression value of two cytolytic markers (GZMA and PRF1) [27], and the geometric mean of these two markers was used to perform the calculations. Tumor mutation burden (TMB), homologous recombination deficiency (HRD), intratumor heterogeneity, nonsilent mutation rate, the number of segments, aneuploidy score, fraction altered, antigen processing and presenting machinery (APM) score, T cell receptor (TCR), transforming growth factor (TGF)‐*b* response, leukocyte fraction, interferon‐gamma (IFNG), interferon‐stimulated genes resistance signature (ISG.RS), and IFNG hallmark gene set (IFNG.GS) were also estimated.

### 2.6 Single‐cell sequencing for annotation of metabolic‐associated clusters

Machine learning pamr validated the metabolic‐associated clusters in glioma cell lines from Cancer Cell Line Encyclopedia (CCLE) and single‐cell sequencing datasets. Cell clustering and dimension reduction were performed using the ‘Seurat' package in r software [17]. After performing the principal component analysis (PCA) using ‘RunPCA', a K‐nearest neighbor was constructed using the ‘FindNeighbors' package. Cells with the highest gene alteration were combined using the ‘FindClusters' package. Dimensionality reduction to visualize the complex expression profiling was performed using ‘t‐SNE'. Malignant cells were identified using the ‘Copykat' package [28]. Non‐malignant cell clusters were annotated using the ‘scCATCH' package [18]. The ‘FindMarkers' package was used in seurat software to identify the significantly differentially expressed genes between malignant cells and non‐malignant glial cells, including astrocyte, oligodendrocytes, and oligodendrocyte precursor cells. Cell–cell interaction analysis was performed using the ‘CellChat' package in r software to infer, analyze, and visualize the different receptor‐ligand signaling expression modules between the three metabolic clusters and the roles of different metabolic clusters in specific pathways [29].

### 2.7 Multiplex immunofluorescence staining

Paraffin sections of glioma tissues collected from Xiangya Hospital, Central South University, were deparaffinized. After antigen retrieval, sections were blocked with 3% H_2_O_2_ and 2% BSA. Different primary antibodies, CD68 (Mouse, 1 : 3000, AiFang Biological, Changsha, China), CD163 (Rabbit, 1 : 3000, Proteintech, Wuhan, China), were sequentially applied, followed by horseradish peroxidase‐conjugated secondary antibody incubation (PV6001, PV6002, ZSGB‐BIO, Beijing, China) and tyramide signal amplification (TSA; Fitc‐TSA, CY3‐TSA and 647‐TSA [Servicebio, Wuhan, China]). After labeling with the human antigens, nuclei were stained with 4′,6‐diamidino2‐phenylindole dihydrochloride (DAPI), and an antifade mounting medium was applied. Stained slides were scanned using the Pannoramic Scanner (3D HISTECH, Budapest, Hungary) to obtain multispectral images. Regarding fluorescence spectra, DAPI glows blue at a UV excitation wavelength of 330–380 nm and emission wavelength of 420 nm, CD163 glows red at an excitation wavelength of 594 nm and emission wavelength of 615 nm, and CD68 glows pink at an excitation wavelength of 608–648 nm and emission wavelength of 672–712 nm. Multispectral images were analyzed, and positive cells were quantified at a single‐cell level by caseviewer (C.V 2.3, C.V 2.0) and pannoramic viewer (P.V 1.15.3) image analysis software.

### 2.8 Cell lines and cell culture

Human glioma cell lines HS683, U251, and U87 were cultured using Dulbecco's modified Eagle's medium (DMEM) supplemented with 10% (FBS). Human microglia cell line HMC3 was cultured using the 1640 complete medium with 10% FBS. HS683, U251, U87, and HMC3 cell lines were purchased from iCell (http://www.icellbioscience.com).

### 2.9 Detection of HA

HA from the culture supernate was detected using the HA ELISA kit (CSB‐E04805h, CUSABIO, Wuhan, China). The standard substance marked with S7 was centrifuged at 6000–10 000 r.p.m. (4028.4‐11 100 *g*) for 30 s and diluted in 1 mL. Seven 1.5‐mL centrifuge tubes were marked with S0–S6. A 250‐μL standard substance was taken from S7 to S6, from S6 to S5, etc., to perform the multiple dilutions. A standard curve was generated according to the diluted S0–S7. The concentration gradient of hyaluronidase was set as 100, 200, 500, 1000 μg·mL^−1^. HA was detected after adding hyaluronidase with different concentrations to the culture supernate.

### 2.10 Cell counting Kit‐8 (CCK8) assay

HS683, U251, and U87 cells were seeded in 96‐well plates at a density of 104 cells per hole. The control and 1000 μg·mL^−1^ hyaluronidase groups of the HS683, U251, and U87 cell lines were cultured for 24, 48, and 72 h, respectively. Each group has three duplicated holes. The absorbance at 450 nm was measured after hatching under at 37 °C and 5% CO_2_.

### 2.11 Clone formation assay

The control and 1000 μg·mL^−1^ hyaluronidase groups of the HS683, U251, and U87 cell lines were digested and plated in 6‐well plates (300 cells per well) and cultured with 5% CO_2_ at 37 °C for 2 weeks. The colonies were fixed with 4% methanol (1 mL per well) for 15 min and stained with 0.5% crystal violet for 30 min at room temperature for photographing.

### 2.12 Wound healing assay

Human glioma cell lines HS683, U251, and U87 were cultured using DMEM with 10% FBS. When the cells grew at a 90% fusion rate, a Marker pen was used to draw horizontal lines evenly, and the cells around the lines were washed out with PBS. The confluent monolayers of cells were observed and photographed after being cultured for 0, 24, and 48 h.

### 2.13 EdU assay

The EdU (5‐ethynyl‐2'‐deoxyuridine) assay was performed according to the manufacturer's instructions (BeyoClick™ EdU Cell Proliferation Kit with Alexa Fluor 488, Shanghai, China). The HS683, U251, and U87 cells were incubated overnight with 50 μL 10 μm EdU medium and then fixed with 4% paraformaldehyde. A 500‐μL Click reaction solution was added for incubation for 30 min. Finally, the cells were incubated with 1 mL 1 × Hoechst 33342 reaction solution for 10 min and observed with a confocal microscope.

### 2.14 Transwell assay for migration

The control and 1000 μg·mL^−1^ hyaluronidase groups of the HS683, U251, and U87 cell lines were digested and resuspended using the serum‐free medium. The density was adjusted to 10^5^ cells·mL^−1^. A 100‐μL cell suspension was added to the upper chamber, and DMEM with 10% FBS was added to the lower chamber. After culturing for 48 h, the upper chamber was washed with PBS twice. The wet swab was used to wipe the cells on the upper layer. The upper chamber was then fixed using acetone and methyl alcohol at a ratio of 1 : 1 for 20 min. After being washed with PBS twice, the upper chamber was stained with 0.5% crystal violet for 5 min for photographing.

### 2.15 Transwell assay for invasion

An 80‐μL aliquot of Matrigel was diluted with 160 μL serum‐free medium on ice; 60 μL diluted Matrigel was then put into each upper chamber and placed in a 37 °C incubator for 60 min for solidification. A 70‐μL aliquot of DMEM was added to the upper chamber to hydrate the basement membrane at 37 °C for 30 min. The rest methodology followed that in the Transwell assay for migration.

### 2.16 Co‐culture assay for Transwell assay

HS683, U251, and U87 cells were cultured in a 6‐well plate at a density of 2 × 10^5^·mL^−1^. The control and 1000 μg·mL^−1^ hyaluronidase groups of HS683, U251, and U87 cells were cultured for 72 h. The control group, 1000 μg·mL^−1^ hyaluronidase group, and 1000 μg·mL^−1^ hyaluronidase + recombinant CHI3L1 (rCHI3L1) protein (R&D Systems, 2599‐CH‐050, Shanghai, China) group of HS683 and U251 cells were cultured for 72 h. HS683, U251, and U87 cells were digested and resuspended using DMEM with 10% FBS at a density of 1 × 10^5^·mL^−1^ and were added to the lower chamber. HMC3 cells were also digested and resuspended at the density of 1 × 10^5^·mL^−1^, and a 100‐μL suspension of HMC3 was added to the upper chamber. Glioma cells and microglia cells were co‐cultured for 72 h. The photographing was conducted as mentioned above.

### 2.17 Co‐culture assay for immunofluorescence staining

The control group and 1000 μg·mL^−1^ hyaluronidase group of HS683 cells in the upper chamber were co‐cultured with HMC3 cells in the lower chamber at a ratio of 1 : 1. After being washed with PBS, HMC3 cells in the lower chamber were incubated with primary antibodies CD68/CD11c and CD68/CD163, respectively. HMC3 cells were subsequently incubated with anti‐mouse and anti‐rabbit IgG secondary antibodies. The cell nucleus of HMC3 cells was stained with DAPI, and incubated HMC3 cells were observed with a microscope.

### 2.18 Western blotting assay

The western blotting assay assessed the expression level of IL‐1, TGF‐β, CHI3L1, PD‐L1, and β‐actin in the control and 1000 μg·mL^−1^ hyaluronidase groups of the HS683 and U251 cells. Anti‐IL‐1 (Rabbit, 1 : 1000, Proteintech, Wuhan, China), anti‐TGF‐β (Rabbit, 1 : 500, Abcam, Cambridge, UK), anti‐CHI3L1 (Rabbit, 1 : 1000, Proteintech, Wuhan, China), anti‐PD‐L1 (Rabbit, 1 : 1000, Abcam, Cambridge, UK), and anti‐β‐actin (Mouse, 1 : 5000, Proteintech, Wuhan, China) were used as the primary antibody. HRP goat anti‐mouse IgG (Mouse, 1 : 5000, Proteintech, Wuhan, China) and HRP goat anti‐rabbit IgG (Rabbit, 1 : 6000, Proteintech, Wuhan, China) were used as the secondary antibody. ECL development was used for visualization.

### 2.19 RNA sequencing

For 105 glioma samples, tumor tissues fixed with RNAstore were collected for sequencing. For U251 and HS683 cells (three in the control group and three in the hyaluronidase group), cells washed with PBS were lysed using Trizol and collected for sequencing. A 1‐μg aliquot of RNA of each sample was prepared for sequencing. The detailed methodology for RNA sequencing was provided in our previous findings [14].

### 2.20 Metabonomics

Per sample, 1 × 10^7^ cells were collected for analyses (three in the control group and three in the hyaluronidase group). The data collection system included Ultra Performance Liquid Chromatography (UPLC; ExionLC AD, https://sciex.com.cn/) and Tandem mass spectrometry (MS/MS; QTRAP®, https://sciex.com.cn/). Quantification of metabolites was performed using the multiple reaction monitoring (MRM) mode.

### 2.21 Statistical analysis

The normality of variables was tested using the Shapiro–Wilk normality test [30]. For the differential gene expression analysis, we used the Benjamini–Hochberg method that converts the *P* values to FDR to identify significant genes [31]. For normally distributed variables, an unpaired Student's *t*‐test was used for comparison between two groups, and the Wilcoxon test was used to compare non‐normally distributed variables. For multiple groups, one‐way analysis of variance was used as a parametric method to compare mean values between groups, and Kruskal–Wallis tests were used as a nonparametric method. All survivorship curves were generated via the r package survminer. All heatmaps were developed based on the r package pheatmap. All statistical analyses were performed in r (https://www.r‐project.org/). All experiments were repeated three times independently. All tests were two‐sided, and *P* values < 0.05 were statistically significant.

## 3 Results

### 3.1 Construction of glioma metabolic pattern

**Fig. 1:**
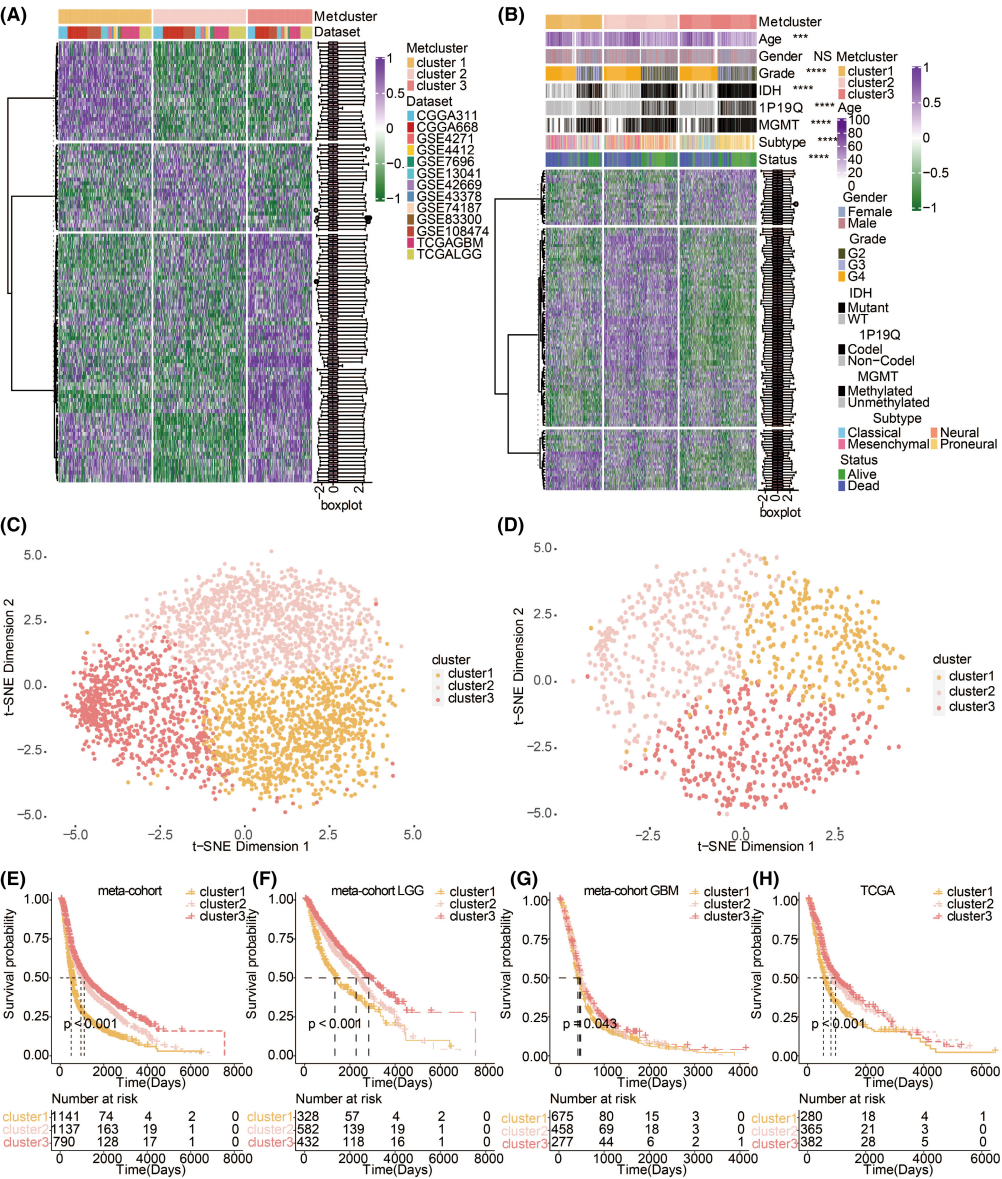
Metabolic phenotypes in gliomas. (A) Unsupervised clustering of metabolism‐relevant pathways for 3068 patients in the meta‐cohort. (B) Unsupervised clustering of metabolism‐relevant pathways for 1027 patients in the TCGA. NS, not statistically significant; *** *P* < 0.001; **** *P* < 0.0001. Statistical analysis was performed using Fisher's exact test. (C) T‐SNE separated the three metabolic clusters in the meta‐cohort. (D) T‐SNE separated the three metabolic clusters in the TCGA. (E) Kaplan–Meier curves for three metabolic clusters of 3068 patients in the meta‐cohort. Log‐rank test, *P* < 0.001. (F) Kaplan–Meier curves for three metabolic clusters of 1342 LGG patients in the meta‐cohort. Log‐rank test, *P* < 0.001. (G). Kaplan–Meier curves for three metabolic clusters of 1410 GBM patients in the meta‐cohort. Log‐rank test, *P* < 0.001. (H) Kaplan–Meier curves for three metabolic clusters of 1027 patients in the TCGA. Log‐rank test, *P* < 0.001.

The flow chart of the whole study is shown in Fig. S1A. The ConsensusClusterPlus package was used to assess clustering stability to determine the optimal cluster number based on smoothest CDF curve (Fig. S1B), which supported three robust subtypes of gliomas in a meta‐cohort (CGGA311, CGGA668, GSE4271, GSE4412, GSE7696, GSE13041, GSE42669, GSE43378, GSE74187, GSE83300, GSE108474, TCGAGBM, and TCGALGG). Consensus clustering was performed for the 3068 patient samples of the 13 included glioma cohorts with the corresponding enrichment score of the 115 metabolic‐related pathways (Fig. 1A). The ConsensusClusterPlus package was also used to assess clustering stability to select the optimal cluster number in TCGA (Fig. S1C). Consensus clustering was performed in the TCGA cohort (1027 patient samples; Fig. 1B), in which three clusters were associated with significantly different clinical characteristics. Among the three clusters, t‐SNE distribution was separated in the meta‐cohort and TCGA alone (Fig. 1C,D). Three metabolic phenotypes were identified by metabolic‐related pathways and exhibited significant differences in the OS in the pan‐gliomas, LGG, GBM from meta‐cohort (Log‐rank test, *P* < 0.001, *P* < 0.001, *P* = 0.043, respectively; Fig. 1E–G). Three metabolic phenotypes with distinct survival outcomes were also identified in TCGA (Log‐rank test, *P* < 0.001; Fig. 1H).

### 3.2 Functional annotation of metabolic clusters

**Fig. 2:**
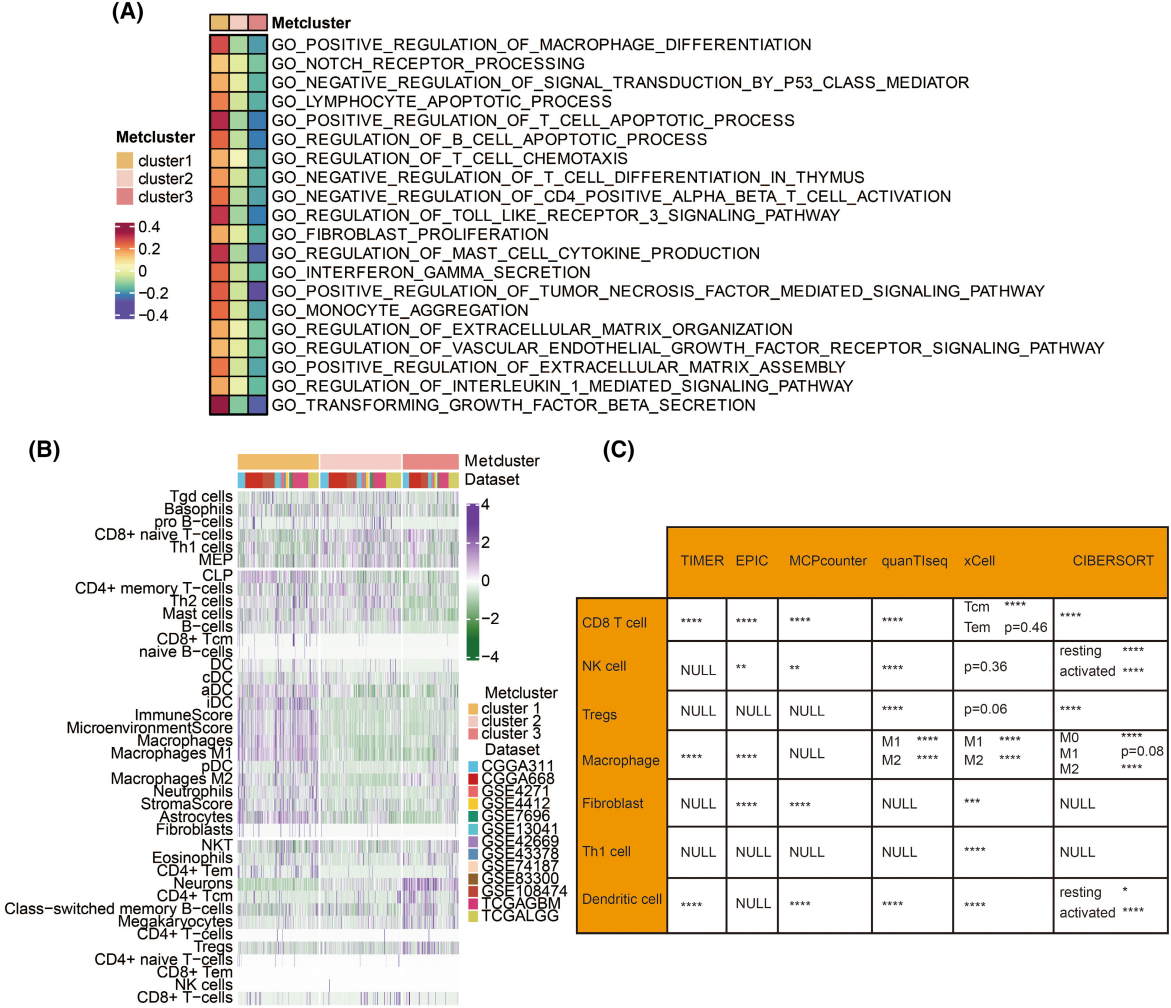
Immune‐related functional annotation of metabolic clusters in meta‐cohort. (A) GSVA of metabolic cluster 1, cluster 2, and cluster 3 in the meta‐cohort (3068 patients) in GO. (B) Heatmap depicting the expression difference of immune cells in three metabolic clusters in the meta‐cohort (3068 patients). (C) Estimating immune cells based on timer, epic, mcpcounter, quantlseq, xcell, and cibersort algorithms. NS, not statistically significant; * *P* < 0.05; ** *P* < 0.01; *** *P* < 0.001; **** *P* < 0.0001. Statistical analysis was performed using Fisher's exact test.

To elucidate the correlation between the immune infiltrating environment and metabolic clusters, 20 of both immune‐related and tumor‐related signaling pathways in GO analysis were identified in the meta‐cohort among cluster 1, cluster 2, and cluster 3 (Fig. 2A). We found that metabolic cluster 1 was positively associated with immunosuppressive pathways, including fibroblast proliferation, positive regulation of macrophage differentiation, lymphocyte apoptotic process, negative regulation of CD4^+^ αβT cell activation, negative regulation of T cell differentiation in the thymus, and regulation of mast cell cytokine production. Metabolic cluster 1 was also positively associated with tumorigenic pathways, including notch receptor processing, regulation of toll‐like receptor 3 signaling pathway, vascular endothelial growth factor (VEGF) receptor signaling pathway, and TGF‐β secretion (Fig. 2A). On the contrary, cluster 3 was negatively associated with immunosuppressive and tumorigenic pathways (Fig. 2A). Consistent with the survival outcomes, cluster 2 was the intermediate state of cluster 1 and cluster 3. Figure 2B shows that the three metabolic clusters exhibited significant differences in immune cell infiltration patterns in meta‐cohort. These differences were reported in multiple immune suppressive cell types, in which macrophages M2, fibroblasts, dendritic cells (DC), and mast cells were more expressed in cluster 1. Immune cell quantification based on timer algorithm, epic algorithm, mcpcounter algorithm, quantlseq algorithm, and cibersort algorithm further revealed significant differences in central immune cell infiltration patterns, including CD8 T cells, NK cells, Tregs, macrophages, fibroblasts, Th1 cells, and dendritic cells among three metabolic clusters (Fig. 2C).

### 3.3 Immune regulatory mechanisms of metabolic clusters

We further explored the potential immune regulatory roles of metabolic clusters. Metabolic cluster 1 was associated more with metastatic and immunosuppressive signatures in the meta‐cohort (Fig. S2A). The intrinsic immune escape mechanism mainly includes three aspects: immune checkpoint molecules, tumor immunogenicity, and antigen presentation capacity [32]. We first explored the association between metabolic clusters and immune checkpoint molecules, which are classified into seven groups: antigen‐presenting, co‐stimulator, co‐inhibitor, cell adhesion proteins, receptors, ligands, and others [25]. Metabolic cluster 1 expressed more immune checkpoints, including CD40, PDCD1LG2, LAG3, PDCD1, ICOSLG, VEGFA, and TGF‐β1 in meta‐cohort (Fig. S2B).

A series of factors associated with tumor immunogenicity was then assessed in TCGA. Metabolic cluster 1 exhibited a higher level of aneuploidy score, intratumor heterogeneity, fraction altered, and nonsilent mutation rate, all of which were significant indicators for genome alteration and tumor malignancy (Figs S2C,D and S3A,B). Metabolic cluster 1 exhibited lower HRD (Fig. S3C). Regarding antigen presentation capacity, metabolic cluster 1 presented a higher APM score, TCR richness, and TCR Shannon (Fig. S3D–F). Stroma signatures, including stromal fraction, leukocyte fraction, and CD8, were significantly higher in metabolic cluster 1 (Fig. S3G–I). Signatures of enforcers of immune homeostasis and tolerance, including TGF‐β response, interferon‐gamma (IFNG), interferon‐stimulated gene resistance signature (ISG.RS), and IFNG hallmark gene set (IFNG.GS), were also significantly higher in metabolic cluster 1 (Figs S2E and S3J–L). The response rates of the tumor to PD‐1 inhibition are reported to be correlated with the TMB [33], CYT [27], and GEP [26]. Metabolic cluster 1 was found to have a higher TMB level (Fig. S2F), CYT level (Fig. S2G), and GEP level (Fig. S2H).

### 3.4 Validating the prognostic value of metabolic‐related pathways

Metabolic‐related pathways also identified three metabolic phenotypes and exhibited significant differences in the OS in eight independent cancer types (Fig. S3M). In addition, consensus clustering was independently performed for CGGA693, CGGA325, and GSE108474 datasets with the corresponding enrichment score of the 115 metabolic‐related pathways (Fig. S4A,C,E, respectively). Consistent with the previous findings, three metabolic phenotypes were identified, and metabolic cluster 1 exhibited worse OS in CGGA693, CGGA325, and GSE108474 (Fig. S4B,D,F, respectively).

Additionally, Monocle was used to reconstruct the cell trace plot mainly containing four branches based on the bulk sequencing data of meta‐cohort, displaying changes in metabolic clusters as pseudotime (a virtual cellular evolution process in which all cells start from the time point of 0) increased (Fig. S5A). Notably, three metabolic clusters were separated at different cell states. The heatmap revealed six diverse evolutionary patterns of metabolic‐related pathways as pseudotime increased in the meta‐cohort (Fig. S5B).

### 3.5 Exploring the characteristics of metabolic clusters based on single‐cell sequencing datasets

**Fig. 3:**
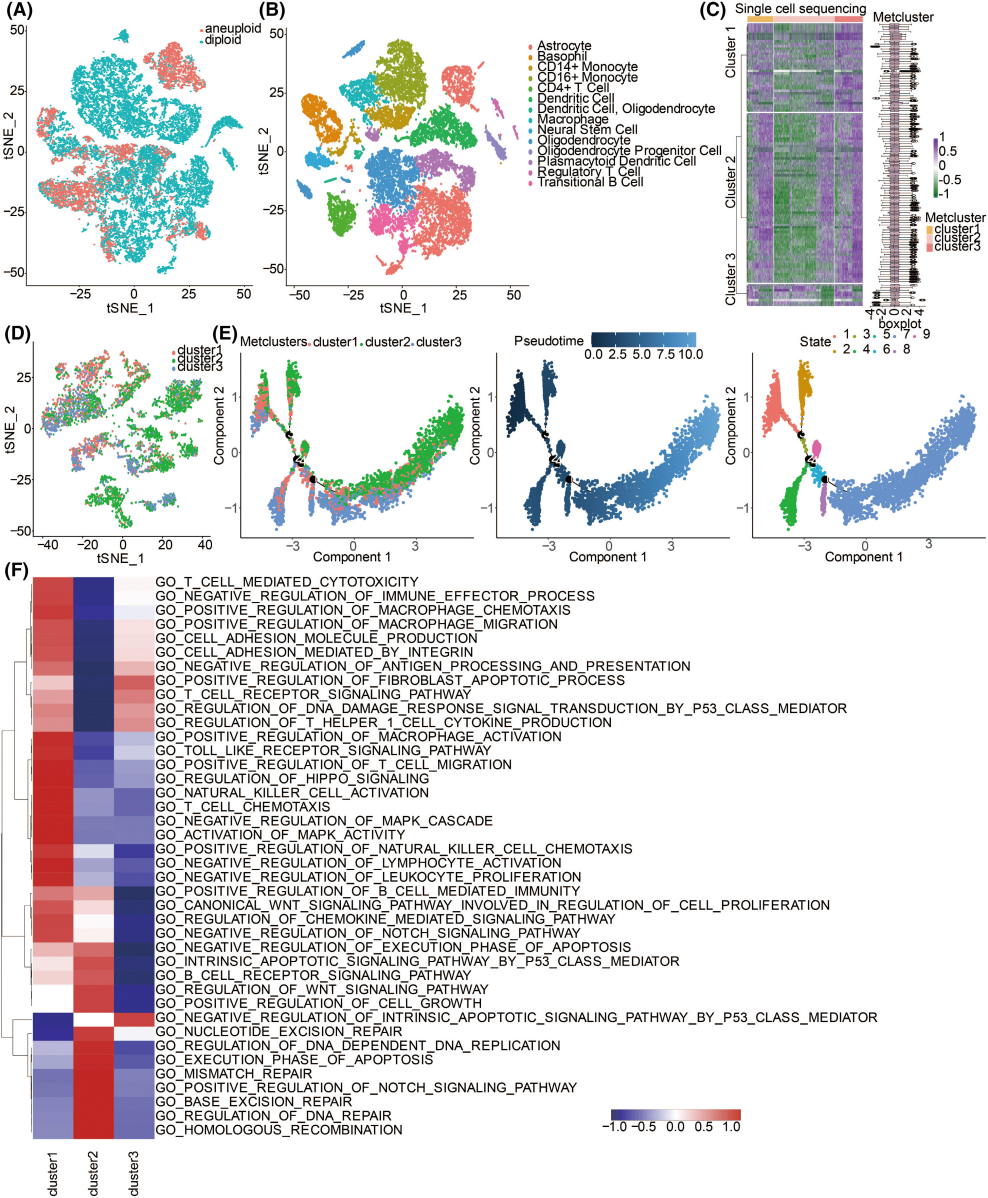
Machine learning validating the clustering results in CCLE and single‐cell sequencing datasets. (A) T‐SNE plot for malignant and non‐malignant cells. (B) T‐SNE plot for the 14 cell clusters annotated in non‐malignant cells. (C) Machine learning pamr validating three metabolic clusters in 33 GBM single‐cell sequencing samples. (D) T‐SNE plot for the three metabolic clusters in malignant cells. (E) The single‐cell trajectory reconstructed by Monocle contains six main branches. Cells are colored based on the cluster (left), pseudotime (middle), and state (right). (F) GSEA results of three metabolic clusters.

Analyzing 33 single‐cell sequencing samples of GBM using t‐SNE for dimension reduction identified malignant and non‐malignant cells (Fig. 3A). Fourteen non‐malignant cell clusters were further annotated: Astrocyte, Basophil, CD14^+^ Monocyte, CD16^+^ Monocyte, CD4^+^ T Cell, Dendritic Cell, Macrophage, Neural Stem Cell, Oligodendrocyte, Oligodendrocyte Progenitor Cell, Plasmacytoid Dendritic Cell, Regulatory T Cell, T Cell, and Transitional B cell (Fig. 3B). Given the robust performance of metabolic‐related pathways in prognosis, we used machine learning pamr to validate three metabolic clusters in a single‐cell sequencing dataset (Fig. 3C), identifying the three corresponding metabolic clusters. Three metabolic clusters in malignant cells were also visualized using a t‐SNE plot (Fig. 3D). GSEA results revealed that cluster 1 was associated with immunosuppressive pathways, including macrophage activation, macrophage migration, macrophage chemotaxis, negative regulation of immune effector process, negative regulation of lymphocyte activation, negative regulation of leukocyte proliferation, and negative regulation of antigen processing and presentation (Fig. 3F). Cluster 1 was also associated with tumorigenic pathways, including toll‐like receptor signaling pathway, activation of MAPK activity, WNT signaling pathway, and regulation of HIPPO signaling (Fig. 3F). In contrast, cluster 3 was associated with immune‐activated pathways, including positive regulation of fibroblast apoptotic process, T cell receptor signaling pathway, and regulation of T helper 1 cell cytokine production (Fig. 3F). Monocle was used to reconstruct the cell trace plot mainly containing six branches based on the single‐cell sequencing data, displaying changes in metabolic clusters as pseudotime increased (Fig. 3E). Notably, three metabolic clusters were clearly separated at different cell states. The heatmap showed that various metabolic signatures were up‐regulated or down‐regulated with an increase in pseudotime (Fig. S6A).

### 3.6 Active cell–cell chat between immune cells and cancerous cells in metabolic clusters

To further explore the differences in intercellular communication between cancerous cells and neighboring non‐GBM brain cells, 16 types of cells were uncovered based on the above single‐cell sequencing analysis, including C1 neoplastic (referring to cancerous cells in cluster 1), C2 neoplastic (referring to cancerous cells in cluster 2), C3 neoplastic (referring to cancerous cells in cluster 3), Astrocyte, Basophil, CD14^+^ Monocyte, CD16^+^ Monocyte, CD4^+^ T Cell, Dendritic Cell, Macrophage, Neural Stem Cell, Oligodendrocyte, Oligodendrocyte Progenitor Cell, Plasmacytoid Dendritic Cell, Regulatory T Cell, T Cell, and Transitional B cell. Given the differentially expressed receptors and ligands of these 16 cell types and the different signaling pathways they were involved in, their roles were classified into receiver, sender, mediator, and influencer. The receiver and sender refer to the receiver and sender of the signaling pathway, respectively, and the mediator and influencer to the mediator and interferer of the signaling pathway, respectively. The 16 cell types were categorized into three patterns regarding their sender role (Fig. S6B). A heatmap depicts the differentially active signaling pathways among three sender patterns (Fig. S6C). Similarly, the 16 cell types were categorized into three patterns regarding their receiver role (Fig. S6D). A heatmap depicts the differentially active signaling pathways among three receiver patterns (Fig. S6E). The dot plot defined targeted cells' incoming (receiver) communication (Fig. S7A) and outcoming (sender) communication (Fig. S7B) patterns. Sankey plot revealed that Astrocyte, Basophil, Neural Stem cell, C1 neoplastic, C2 neoplastic, and C3 neoplastic correlated with receiver and sender signaling pathways in pattern 3 (Fig. S7C,D, respectively).

### 3.7 *In vitro* validation of the metabolic phenotypes

**Fig. 4:**
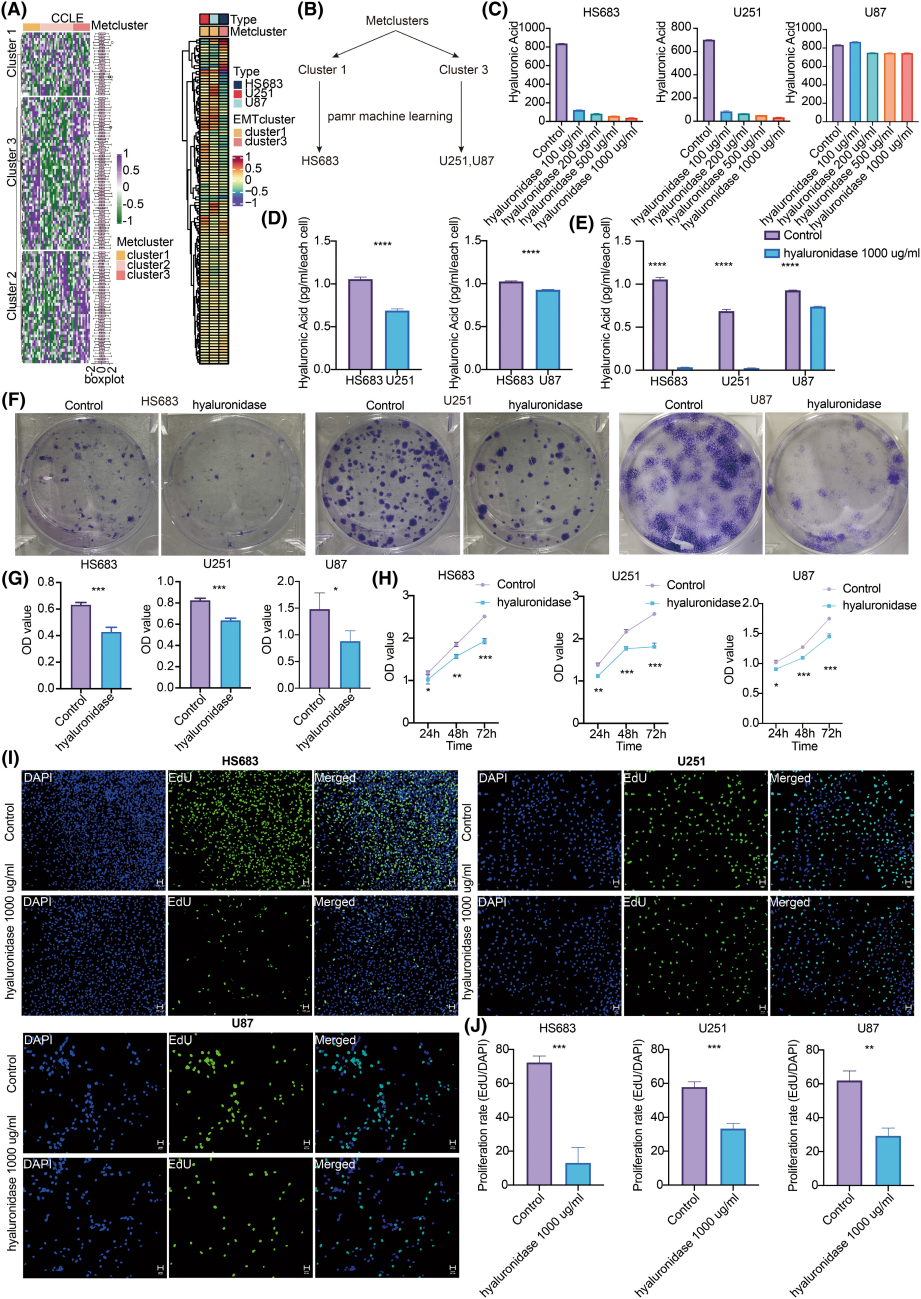
HA in the proliferation of gliomas. (A) Machine learning pamr validating three metabolic clusters in CCLE. (B) Diagram of the machine learning for CCLE. (C) ELISA for detection of HA in HS683, U251, and U87 cell lines treated with hyaluronidase of different concentrations. (D) Statistical analysis of HA in HS683, U251, and U87 cell lines. Statistical analysis was performed using an unpaired Student's *t*‐test. (E) Statistical analysis of HA in the control and hyaluronidase groups of HS683, U251, and U87 cell lines. Statistical analysis was performed using an unpaired Student's *t*‐test. (F) Clone formation assay in HS683, U251, and U87 cell lines. (G) Statistical analysis of clone formation assay in the control and hyaluronidase groups in HS683, U251, and U87 cell lines. Statistical analysis was performed using an unpaired Student's *t*‐test. (H) CCK8 assay in HS683, U251, and U87 cell lines. (I) EdU assay in HS683, U251, and U87 cell lines. Scale bars: 100 and 50 μm, respectively. (J) Statistical analysis of EdU assay in the control and hyaluronidase groups in HS683, U251, and U87 cell lines. Statistical analysis was performed using an unpaired Student's *t*‐test. Data are represented as mean ± SD. The results shown are representative of three independent experiments. * *P* < 0.05; ** *P* < 0.01; *** *P* < 0.001; **** *P* < 0.0001.

**Fig. 5:**
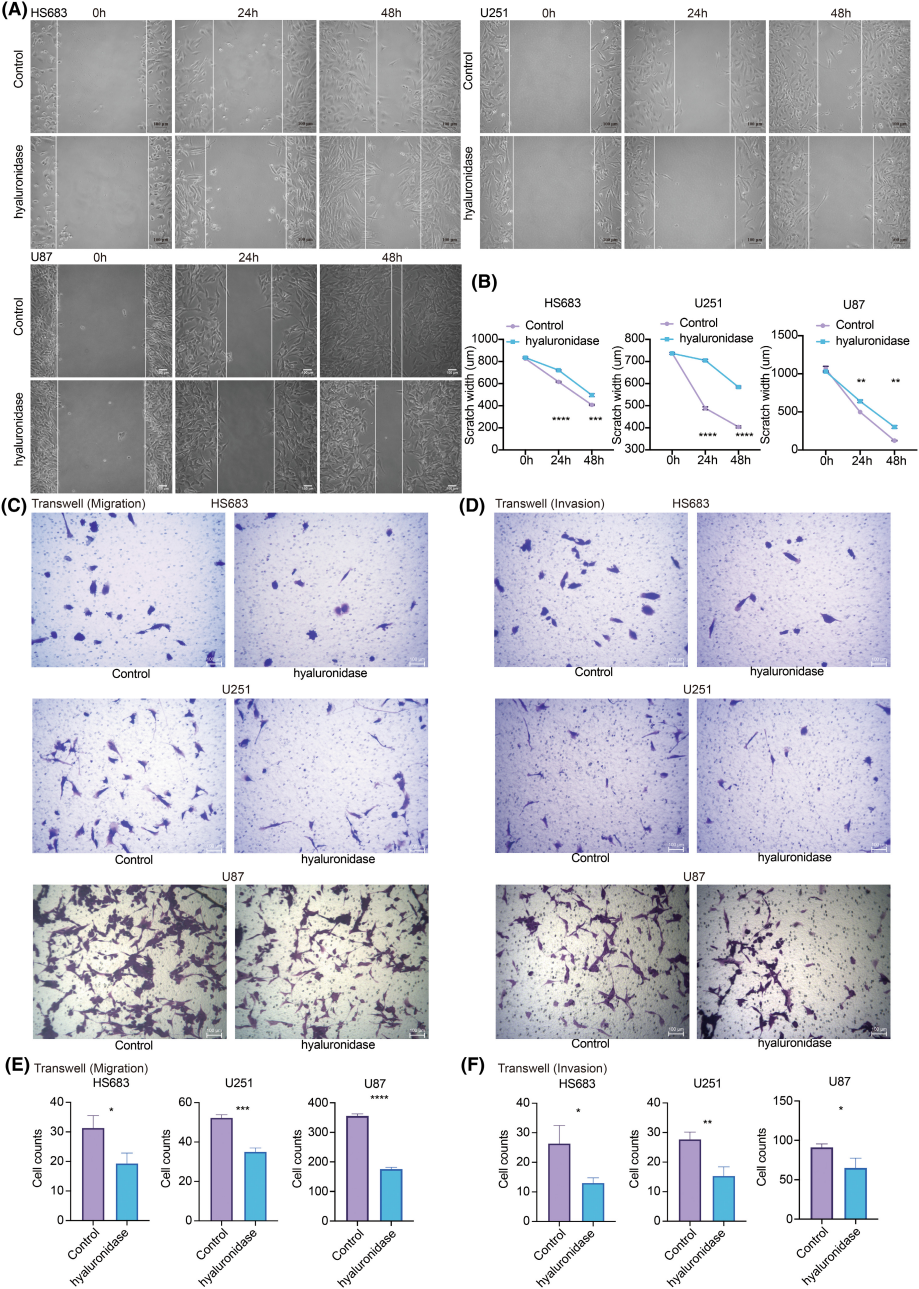
HA in the progression and invasion of gliomas. (A) Wound healing assay in HS683, U251, and U87 cell lines. Scale bar: 100 μm. (B) Statistical analysis of scratch width in the control and hyaluronidase groups of HS683, U251, and U87 cell lines. Statistical analysis was performed using an unpaired Student's *t*‐test. (C) Transwell assay for migration in the control and hyaluronidase groups of HS683, U251, and U87 cell lines. Scale bar: 100 μm. (D) Transwell assay for invasion in the control and hyaluronidase groups of HS683, U251, and U87 cell lines. Scale bar: 100 μm. (E) Statistical analysis of Transwell assay for migration in the control and hyaluronidase groups in HS683, U251, and U87 cell lines. Statistical analysis was performed using an unpaired Student's *t*‐test. (F) Statistical analysis of Transwell assay for invasion in the control and hyaluronidase groups in HS683, U251, and U87 cell lines. Statistical analysis was performed using an unpaired Student's *t*‐test. Data are represented as mean ± SD. The results shown are representative of three independent experiments. * *P* < 0.05; ** *P* < 0.01; *** *P* < 0.001; **** *P* < 0.0001.

Glycosaminoglycan ranked first in cluster 1 with the highest HR value (Table S3). Interaction of glycosaminoglycan with growth factors, growth factor receptors, and cytokines has been implicated in tumor growth, progression, and metastasis [34]. As a vital component of glycosaminoglycan, HA has been widely studied. Therefore, the expression differences of HA in metabolic clusters were explored. Based on the predicted cell lines from CCLE using machine learning pamr in Fig. 4A, HS683 was identified in cluster 1, and U251 and U87 were identified in cluster 3 (Fig. 4B). HA concentration decreased as hyaluronidase concentration increased in HS683, U251, and U87 (Fig. 4C), in which 1000 μg·mL^−1^ hyaluronidase had the highest efficiency degrading HA. Statistical significance was observed in HS683, U251, and U87 regarding the HA concentration in the control and hyaluronidase‐treated groups (Fig. 4E). As expected, HS683 from cluster 1 had a significantly higher HA level than U251 and U87 from cluster 3 (Fig. 4D). Cell clone formation assay proved that hyaluronidase inhibited the clonality of glioma cells (Fig. 4F,G). CCK8 assay revealed that the control group had suppressed cell proliferation compared with the hyaluronidase‐treated group in HS683, U251, and U87 (Fig. 4H). EdU assay further showed that hyaluronidase decreased the proliferation ability of HS683, U251, and U87 cells (Fig. 4I,J). In the wound healing assay, the migration ability of HS683, U251, and U87 cells decreased after treatment with hyaluronidase for 24 and 48 h (Fig. 5A,B). Moreover, HS683, U251, and U87 cells had a statistically significant (Fig. 5E,F) decreased ability to migrate (Fig. 5C) and invade (Fig. 5D) in the hyaluronidase‐treated group. These findings supported that HA mediates glioma proliferation, progression, and invasion.

### 3.8 HA mediates the infiltration and migration of macrophages

**Fig. 6:**
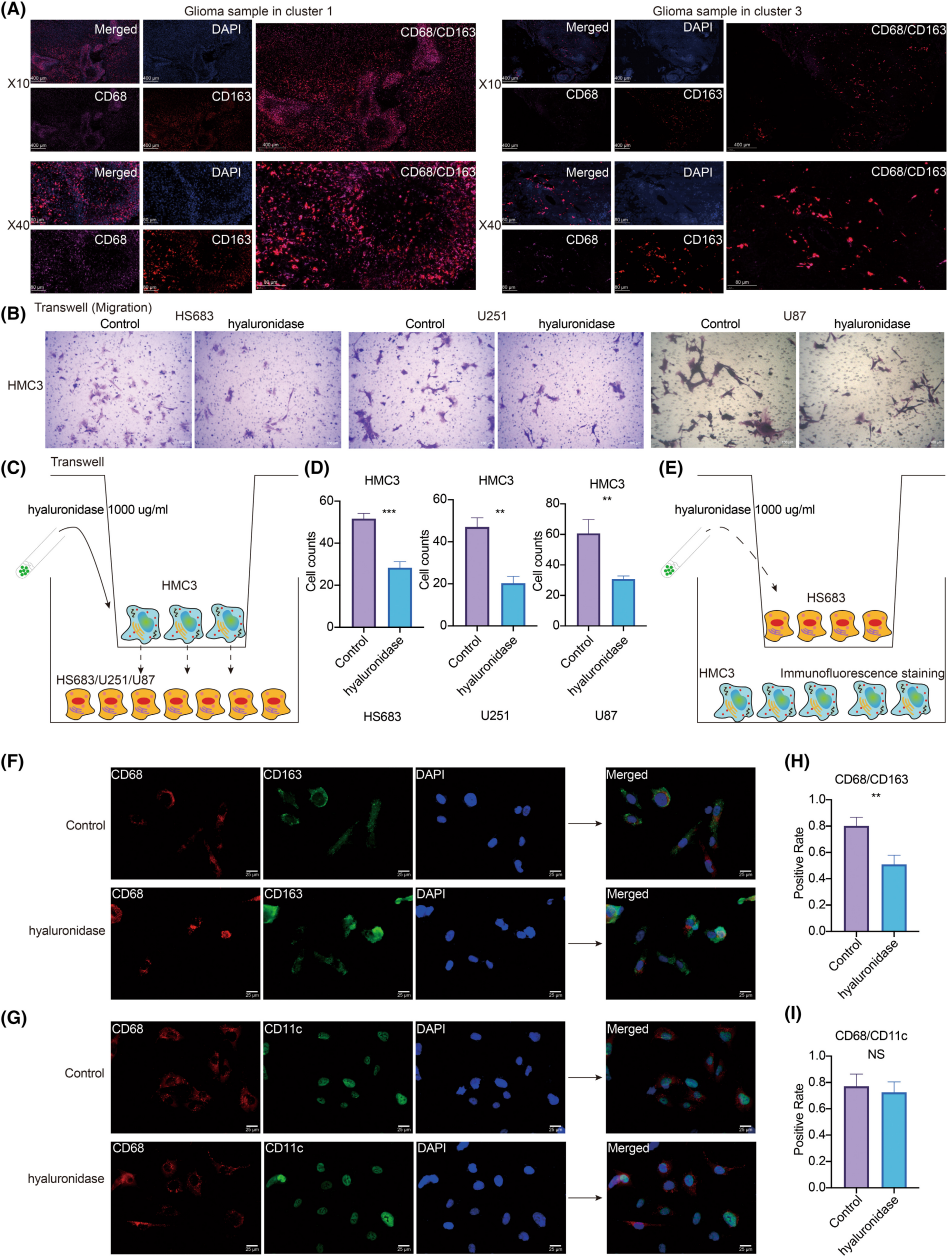
HA in the infiltration, migration, and polarization of macrophages. (A) Multiplex immunofluorescence staining of CD68 (pink), CD163 (red), and DAPI (blue) on glioma whole tissue section (10× and 40×). Scale bars: 400 and 80 μm, respectively. (B) Transwell assay for migration of HMC3 in the control group and hyaluronidase group after co‐culture of HMC3 and glioma cell lines, HS683, U251, and U87. Scale bar: 100 μm. (C) Flow diagram of the co‐culture system for Transwell assay. (D) Statistical analysis of Transwell assay for migration of HMC3 after co‐culture of HMC3 and glioma cell lines, HS683, U251, and U87. Statistical analysis was performed using an unpaired Student's *t*‐test. (E) Flow diagram of the co‐culture system for immunofluorescence staining. (F) Immunofluorescence staining of CD68 and CD163 in the control and hyaluronidase groups after co‐culture of HMC3 and HS683. Scale bar: 25 μm. (G). Immunofluorescence staining of CD68 and CD11c in the control and hyaluronidase groups after co‐culture of HMC3 and HS683. Scale bar: 25 μm. (H). Statistical analysis of immunofluorescence staining of CD68 and CD163. Statistical analysis was performed using an unpaired Student's *t*‐test. (I). Statistical analysis of immunofluorescence staining of CD68 and CD11c. Statistical analysis was performed using an unpaired Student's *t*‐test. Data are represented as mean ± SD. The results shown are representative of three independent experiments. NS, not statistically significant; ** *P* < 0.01; *** *P* < 0.001.

According to the results from bulk sequencing and single‐cell sequencing analysis, macrophages were more infiltrated in glioma samples from cluster 1. Consistent with this finding, a more prominent overlap in CD68^+^ and CD163^+^ (cytoplasm and cell membrane) cell distribution was observed in tissue sections from cluster 1 than in tissue sections from cluster 3 in the Xiangya cohort (Fig. 6A). To further explore the role of HA in the infiltration of macrophages, HMC3 cells were co‐cultured with HS683, U251, and U87 cells. In the hyaluronidase‐treated group, HMC3 cells had decreased migration ability (Fig. 6B) with statistical significance in the HS683 group, the U251 group, and the U87 group (Fig. 6D). The co‐culture system for the Transwell assay is illustrated in Fig. 6C. The co‐culture system for immunofluorescence staining is shown in Fig. 6E. In the hyaluronidase‐treated group of HS683 cells, HMC3 cells had a statistically significant (Fig. 6H) decreased expression of M2 polarization markers CD68/CD163 (Fig. 6F). The expression of M1 polarization markers CD68/CD11c was not significantly different between the control group and the hyaluronidase‐treated group (Fig. 6G,I).

### 3.9 RNA sequencing of HS683 and U251 cells

**Fig. 7:**
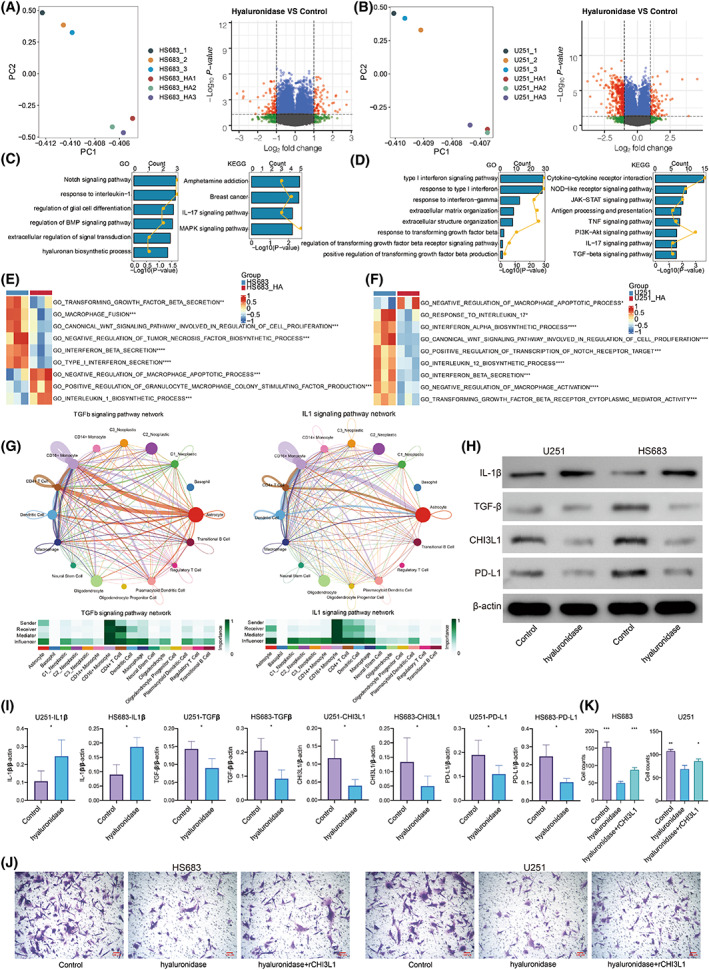
RNA sequencing in HS683 and U251 cells. (A) Principal components analysis of HS683 samples. Volcano plot of the differentially expressed genes between the control and hyaluronidase groups of HS683 cells. (B) Principal components analysis of U251 samples. Volcano plot of the differentially expressed genes between the control and hyaluronidase groups of U251 cells. (C) GO and KEGG enrichment analysis for differentially expressed genes in HS683 cells. (D) GO and KEGG enrichment analysis for differentially expressed genes in U251 cells. (E) GSVA of GO terms for differentially expressed genes in HS683 cells. (F) GSVA of GO terms for differentially expressed genes in U251 cells. (G) The cellular interaction network for the relationship among three metabolic clusters and infiltration of immune cells regarding IL‐1b and TGF‐β. (H) Western blotting results of IL‐1b, TGF‐β, CHI3L1, and PD‐L1 in HS683 and U251 cell lines. (I) Statistical analysis of western blotting results of IL‐1b, TGF‐β, CHI3L1, and PD‐L1 in HS683 and U251 cell lines. Statistical analysis was performed using an unpaired Student's *t*‐test. (J) Transwell assay for migration in the control, hyaluronidase, and hyaluronidase + rCHI3L1 groups of HS683 and U251 cell lines. Scale bar: 100 μm. (K) Statistical analysis of Transwell assay for migration in the control, hyaluronidase, and hyaluronidase + rCHI3L1 groups in HS683 and U251 cell lines. Statistical analysis was performed using an unpaired Student's *t*‐test. Data are represented as mean ± SD. The results shown are representative of three independent experiments. * *P* < 0.05; ** *P* < 0.01; *** *P* < 0.001.

To explore the potential signaling pathways involved in the regulation of HA on macrophages, RNA sequencing analysis was performed on the HS683 and U251 cells (Fig. 7A,B, respectively). CHI3L1 was found to be the top‐ranked DEG with statistical significance. CHI3L1 has been reported to significantly facilitate the recruitment, migration, and polarization of macrophages [35]. We hypothesized that HA might potentially regulate macrophage activity through activating CHI3L1. GO and KEGG enrichment analysis based on the DEG between two groups in HS683 (Fig. 7C) and U251 cells (Fig. 7D) showed that notch signaling pathway, response to interleukin‐1, response to TGF‐β, and IL‐17 signaling pathway were significantly active. GSVA of GO terms also proved that IL‐1, TGF‐β, and regulation of macrophage signaling pathways were differentially active between two groups in HS683 (Fig. 7E) and U251 cells (Fig. 7F). In addition, based on the delineated cell communication patterns in TME of GBM, tumor cells from cluster 1 strongly interacted with macrophages via IL‐1 and TGF‐b signaling pathways (Fig. 7G). IL‐1 was reported to inhibit the expression of CHI3L1 [36], whereas TGF‐β was said to facilitate the expression of CHI3L1 [37]. Thus, HA was hypothesized to mediate the activity of CHI3L1 by suppressing IL‐1 and enhancing TGF‐β signaling pathways. In line with this hypothesis, the expression level of IL‐1 increased in the hyaluronidase‐treated group in HS683 and U251 (Fig. 7H) with statistical significance (Fig. 7I). In contrast, the expression level of TGF‐β decreased in the hyaluronidase‐treated group in HS683 and U251 (Fig. 7H) with statistical significance (Fig. 7I). The expression level of CHI3L1 decreased in the hyaluronidase‐treated group in HS683 and U251 (Fig. 7H) with statistical significance (Fig. 7I). In addition, the expression level of PD‐L1 decreased in the hyaluronidase‐treated group in HS683 and U251 (Fig. 7H) with statistical significance (Fig. 7I). To further establish whether HA mediates the infiltration of macrophages by affecting CHI3L1, HMC3 cells were again co‐cultured with HS683 cells and U251 cells. Compared with the hyaluronidase‐treated group, HMC3 cells in the hyaluronidase + rCHI3L1‐treated group had increased migration ability (Fig. 7J) with statistical significance in both the HS683 group and the U251 group (Fig. 7K).

### 3.10 Metabolomics of HS683 and U251 cells

**Fig. 8:**
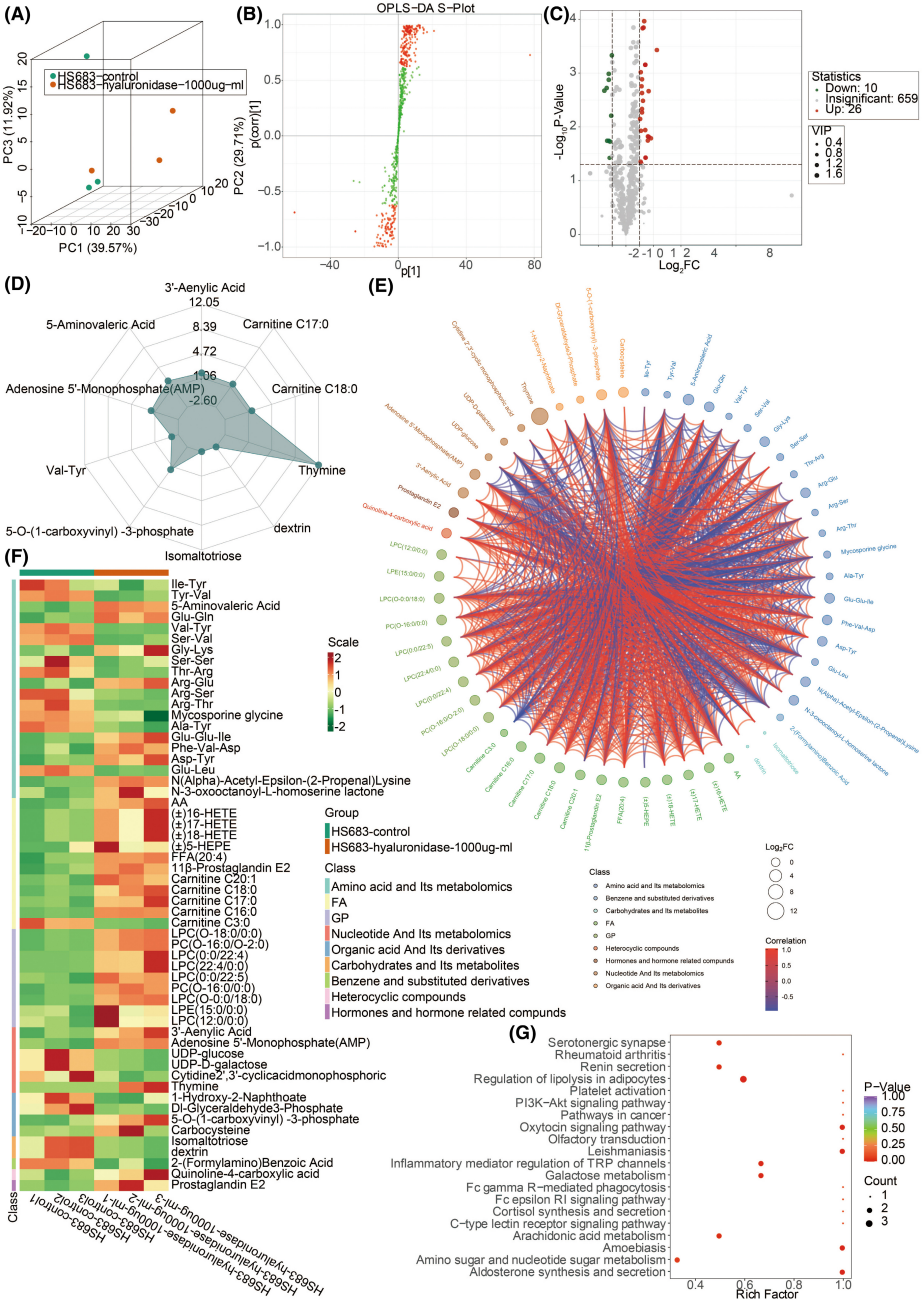
Metabolomics in HS683 cells. (A) 3D plot for principal components analysis of HS683 samples. (B) S‐plot of orthogonal projection on latent structure‐discriminant analysis of HS683 samples. The red dot indicates the VIP value of metabolites larger than 1, while the green shows the VIP value of metabolites less than 1. (C) Volcano plot of the differentially expressed metabolites between the control group and hyaluronidase group of HS683 cells. (D) Radar plot showing the top 10 differentially expressed metabolites. (E) Chordal graph showing the interconnection of the differentially expressed metabolites. (F) Heatmap of the differentially expressed metabolites based on unit variance scaling. (G) KEGG enrichment analysis of the differentially expressed metabolites. The rich factor is the ratio of the differentially expressed metabolites and the total defined metabolites in the corresponding pathway. The results shown are representative of three independent experiments.

To comprehensively define the role of the HA in the tumor immune microenvironment of gliomas, metabolomics was performed on the HS683 and U251 cells. The PCA results of the six samples in HS683 and U251 cells are shown in Figs 8A and S8A, respectively. To better extract the principal information for differential analysis, the Orthogonal Projection on Latent Structure‐Discriminant Analysis (OPLS‐DA) was performed, and the S‐plot based on the Variable Importance in Projection (VIP) value of metabolites was generated (Figs 8B and S8B). The differentially expressed metabolites between the control and hyaluronidase groups in HS683 and U251 cells were shown in Figs 8C and S8C, respectively. The top 10 differentially expressed metabolites in HS683 and U251 cells are shown in Figs 8D and S8D, respectively. The interconnection of the differentially expressed metabolites in HS683 and U251 cells is shown in Figs 8E and S8E, respectively. The differentially expressed metabolites based on Unit Variance Scaling in HS683 and U251 cells are shown in Figs 8F and S8F, respectively; fatty acids (FA) and glycerophospholipid (GP) were more active in the hyaluronidase group in HS683 cells.

In contrast, carbohydrates and their metabolites, FA, GP, co‐enzymes and vitamins, hormones and hormone‐related compounds, bile acids, and sphingolipids (SL) were more active in the hyaluronidase group in U251 cells. Specifically, glycolytic metabolism was less engaged in the control group, whereas metabolite arginine was more active in the control group. KEGG enrichment analysis of the differentially expressed metabolites showed that HA was more involved in the PI3K‐Akt signaling pathway, Fc gamma R‐mediated phagocytosis, and cancer‐related pathways in HS683 cells (Fig. 8G). HA was also more engaged in the PI3K‐Akt signaling pathway in U251 cells (Fig. S8G). The functional annotation of the differentially expressed metabolites in HS683 and U251 cells is shown in Figs S9A and S10A, respectively. The metabolic change of the KEGG pathways based on DA score in HS683 and U251 cells is shown in Figs S9B and S10B, respectively. The identified KEGG pathways were classified in HS683 and U251 cells in Figs S9C and S10C, respectively. The Metabolite Set Enrichment Analysis (MSEA) based on the differentially expressed metabolites in HS683 and U251 cells is shown in Figs S9D and S10D, respectively. The top Human Metabolome Database (HMDB) primary pathways based on the differentially expressed metabolites in HS683 and U251 cells are shown in Figs S9E and S10E, respectively.

## 4 Discussion

TME plays a central role in facilitating the proliferation and progression of tumor cells. Previous studies have demonstrated that the metabolism of immune cells influences their differentiation and function. The interplay of multiple factors within the TME profoundly influences the metabolic activities of immune infiltrating cells and tumor cells [38, 39]. A previous study has managed to stratify LGG patients into distinct subtypes based on metabolic expression profiling [40]. However, they have mainly focused on the prognostic value of the metabolic subtypes. No complex and comprehensive interconnection between metabolism and TME in gliomas based on bulk and single‐cell sequencing analysis has been fully elucidated. In this study, metabolic‐related pathways efficiently classified samples from glioma and pan‐cancer datasets, demonstrating the robustness and potential of metabolic‐related pathways despite the tumor heterogeneity. Glioma patients were classified into three groups, metabolic cluster 1, cluster 2, and cluster 3, to study the relationship between tumor metabolic patterns and immune infiltrating cells within TME.

Three metabolic clusters exhibited different survival outcomes and biological functions. The isocitrate dehydrogenase (IDH) mutation confers better survival outcomes in glioma patients [41]. The co‐deletion of chromosomes 1p and 19q is also a joint event predicting better survival outcomes in gliomas [42]. The O6‐methylguanine‐DNA methyltransferase (MGMT) methylated glioma is more sensitive to alkylated drugs [42]. Human gliomas have four different molecular subclasses: classic (CL) and mesenchymal (ME) with more aggressive behavior, and pro‐neural (PN) and neural (NE) with relevant benign behavior [43]. Consistently, metabolic cluster 1 was associated more with IDH wild type, 1p/19q non‐co‐deletion, MGMT un‐methylated, and CL/ME subtypes of gliomas. Metabolic cluster 1 was enriched in genes involved in signaling pathways related to tumorigenic and immunosuppressive processes and was associated with poor survival. Metabolic cluster 3 was significantly associated with the immune‐activated function and predicted better survival. In addition, all immunosuppressive cells, including M2 macrophages, DC, mast cells, and fibroblasts, were more highly expressed in metabolic cluster 1. Subsequently, the potential immune characteristics of metabolic clusters were summarized and underlined. Immune checkpoint molecules including CD40, PDCD1LG2, LAG3, PDCD1, ICOSLG, VEGFA, and TGFB1 were observed more in metabolic cluster 1, indicating an immunosuppressive microenvironment [44]. Moreover, metabolic cluster 1 prominently participated in regulating immunomodulators for tumor immunogenicity and antigen presentation capacity. Metabolic cluster 1 was also detected with higher Intratumor Heterogeneity, a diagnostic phenotype with a greater malignancy from cancer [45].

Additionally, metabolic cluster 1 had distinct biological characteristics regarding stroma signatures such as TGF‐β response, leukocyte fraction, and ISG.RS. These stroma signatures have previously been proved to facilitate the immune escape of cancer [46]. The above findings suggested a novel orientation for the inclusion of metabolic clusters as indicators of immunosuppression. Further, metabolic cluster 1 correlated with higher levels of TMB, CYT, and GEP, which are valuable markers in predicting immunotherapeutic response. Thus, glioma patients from metabolic cluster 1 were more likely to benefit from immunotherapy.

Given that three metabolic clusters were separated based on the cell trace reconstructed by Monocle in bulk sequencing datasets, we further tried to elucidate the characteristics of metabolic clusters in single‐cell sequencing datasets. Consistently, the three corresponding metabolic clusters developed and verified by machine learning pamr were separated at different cell states. Consistent with the findings in bulk sequencing datasets, metabolic cluster 1 was related to the tumorigenic and immunosuppressive processes, whereas metabolic cluster 3 was associated with the immune‐activated function. It should be noted that TME consists of diverse adaptive and innate immune cells that perform both pro‐tumorigenic and anti‐tumorigenic functions [32].

To determine the role of metabolism in the interaction between glioma cells and macrophages, we focused on glycosaminoglycan, ranked first in cluster 1 with the highest HR value. As the primary component of glycosaminoglycan, our *in vitro* analysis proved that HA critically mediated glioma proliferation, progression, and invasion. Besides, glioma cells regulated the migration and recruitment of macrophages by releasing HA. Based on the Xiangya cohort, macrophage markers CD68 and CD163 were more abundantly expressed in glioma samples from cluster 1. Consistent with this finding, HS683, a glioma cell line identified in cluster 1, was found to regulate the M2 polarization of macrophages by releasing HA. Subsequently, as two signaling pathways involved in the interaction between GBM cells and macrophages, IL‐1 and TGF‐β signaling pathways were significantly differentially active in the control and hyaluronidase groups. HA was further found to suppress the expression of IL‐1 and enhance the expression of TGF‐β.

Additionally, HA was revealed to enhance the expression of CHI3L1. IL‐1 was reported to inhibit the expression of CHI3L1 [36], whereas TGF‐β was said to facilitate the expression of CHI3L1 [37]. Moreover, CHI3L1 has been reported to play a significant role in the activity of macrophages [35]. Consistently, HA may suppress the expression of IL‐1 and enhance the expression of TGF‐β, indicating that HA could potentially regulate the IL‐1/CHI3L1 and TGF‐β/CHI3L1 axis. HA was further revealed to directly stimulate the migration of macrophages by enhancing the expression of CHI3L1. HA also increased the expression of PD‐L1 in HS683 and U251 cells. The previous study has also demonstrated that CHI3L1 could promote the expression of PD‐L1 [47], which further proved the immunosuppressive role of HA in the tumor microenvironment of gliomas.

To further determine the role of the HA in the metabolism of the tumor microenvironment of gliomas. The metabolomics analyses revealed that 5_Aminovaleric Acid was the joint top‐ranked differentially expressed metabolite between the control and hyaluronidase groups in U251 and HS683 cells. HA was found to influence fatty acids (FA) and glycerophospholipid (GP) in HS683 cells, while influencing carbohydrates and their metabolites, FA, GP, coenzymes and vitamins, hormones, and hormone‐related compounds, bile acids, and sphingolipids (SL) in U251 cells. Specifically, HA was found to significantly affect the expression of metabolite arginine, which is used by M2 macrophages as a substrate for Arginase1 that critically mediates T cell suppression [10, 48]. Moreover, HA could limit glucose, favoring M2 macrophages over M1 macrophages [10, 48]. Functional annotation of differentially expressed metabolites revealed that HA was more involved in the PI3K‐Akt signaling pathway, Fc gamma R‐mediated phagocytosis, and pathways in cancer in HS683 cells. At the same time, HA was more engaged in the PI3K‐Akt signaling pathway in U251 cells.

## 5 Conclusions

This study developed three metabolic clusters based on metabolic‐related pathways that enabled us to comprehensively explore the interconnection between metabolism and TME cells in individual glioma patients. Metabolic clusters showed differences in HA that significantly promoted the proliferation, migration, invasion of GBM, and the infiltration and recruitment of macrophages through the IL‐1/CHI3L1 and TGF‐β/CHI3L1 axis. HA also regulated the expression of PD‐L1 and facilitated an immunosuppressive microenvironment. Therefore, the metabolic clusters established here could help in the development of effective therapeutic strategies.

## Conflict of interest

The authors declare no conflict of interest.

## Author contributions

HZ, QC, NZ, ZW, ZD, XZ, WW, WY, JZ, PL, ZL, LZ, XL, SF, and ZL designed and drafted the manuscript. HZ and QC wrote the figure legends and revised the article. ZD and NZ conducted the data analysis. HZ did the *in vitro* experiments. ZL and QC supervised the study. All authors read and approved the final article.

### Peer review

The peer review history for this article is available at https://publons.com/publon/10.1002/1878‐0261.13315.

## Supporting information


**Fig. S1.** Development of metabolic phenotypes in gliomas.
**Fig. S2.** Immune characteristics of metabolic clusters.
**Fig. S3.** Immune characteristics and prognostic value of metabolic clusters.
**Fig. S4.** Construction of three metabolic clusters in external datasets.
**Fig. S5.** Reconstruction of a single cell trajectory.
**Fig. S6.** Functional annotation of metabolic clusters based on single‐cell sequencing datasets.
**Fig. S7.** Cellular interaction network.
**Fig. S8.** Metabolomics sequencing in U251 cells.
**Fig. S9.** Functional annotation of the differentially expressed metabolites in HS683 cells.
**Fig. S10.** Functional annotation of the differentially expressed metabolites in U251 cells.

**Table S1.** Basic information of 13 included cohorts.

**Table S2.** Basic information of included patients.

**Table S3.** Metabolic pathways in cluster 1.

## Data Availability

All data used in this work can be acquired from the Gene Expression Omnibus (GEO; https://www.ncbi.nlm.nih.gov/geo/), the Cancer Genome Atlas (TCGA) datasets (https://xenabrowser.net/), the Chinese Glioma Genome Atlas (CGGA) datasets (http://www.cgga.org.cn/).
