# Supplementary material for: Hyaluronic acids mediate the infiltration, migration, and M2 polarization of macrophages: evaluating metabolic molecular phenotypes in gliomas
Source: Mol Oncol. 2022 Oct 10;16(22):3927–48. doi: 10.1002/1878-0261.13315 (PMC9718117; doi:10.1002/1878-0261.13315)
Supplement: Supplementary file 1 — Fig. S1. Development of metabolic phenotypes in gliomas. Fig. S2. Immune characteristics of metabolic clusters. Fig. S3. Immune characteristics and prognostic value of metabolic clusters. Fig. S4. Construction of three metabolic clusters in external datasets. Fig. S5. Reconstruction of a single cell trajectory. Fig. S6. Functional annotation of metabolic clusters based on single‐cell sequencing datasets. Fig. S7. Cellular interaction network. Fig. S8. Metabolomics sequencing in U251 cells. Fig. S9. Functional annotation of the differentially expressed metabolites in HS683 cells. Fig. S10. Functional annotation of the differentially expressed metabolites in U251 cells. [file MOL2-16-3927-s001.docx]

**Supplementary Figures**

**
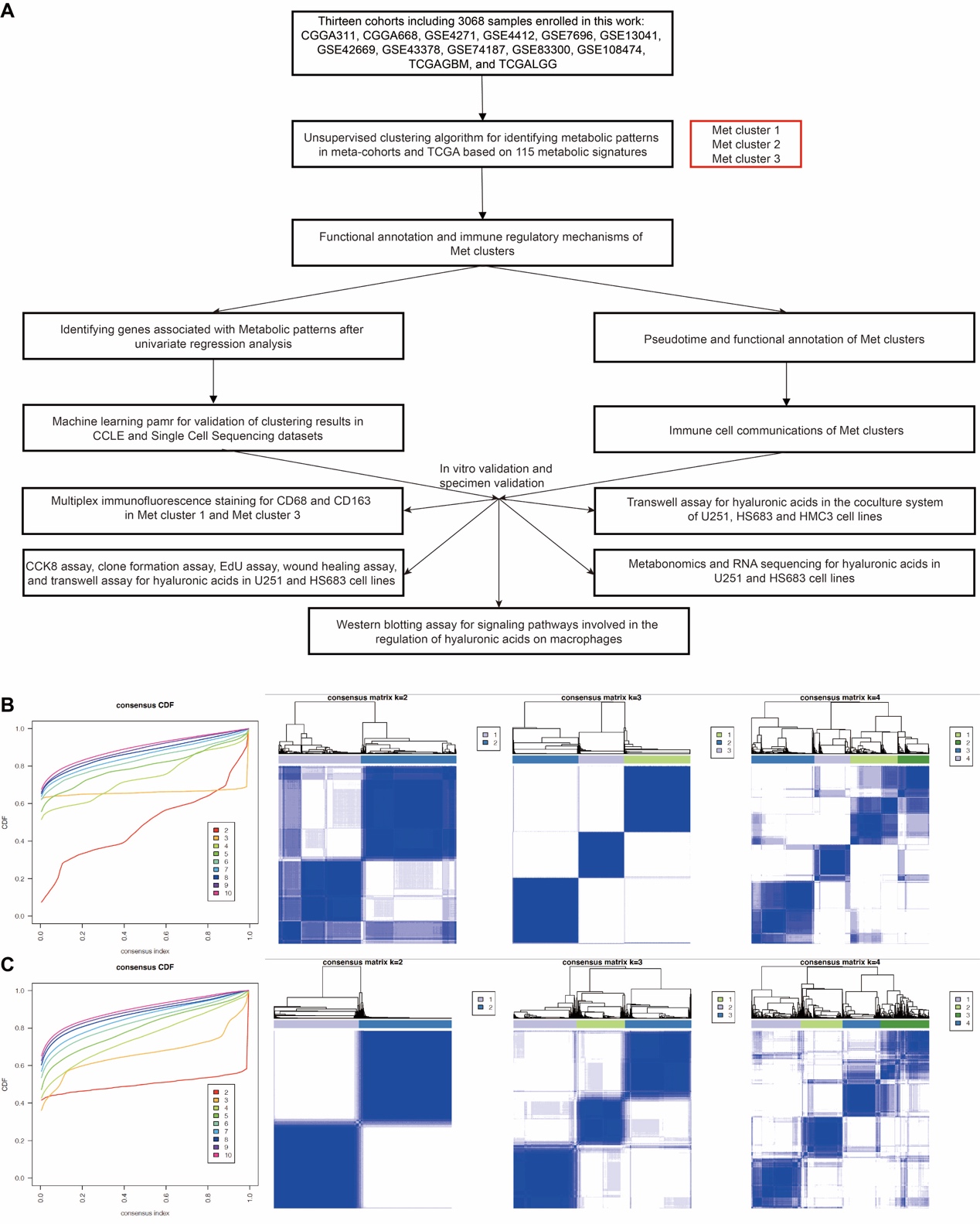
**

**Figure S1. Development of metabolic phenotypes in gliomas. A**. Flow chart of this study. **B**. Consensus clustering cumulative distribution function (CDF) for k=2 to 10 in meta-cohort. Consensus matrices of meta-cohort for each k (k = 2–4), displaying clustering stability using 1000 iterations of hierarchical clustering. **C**. Consensus clustering cumulative distribution function (CDF) for k=2 to 10 in TCGA. Consensus matrices of TCGA for each k (k = 2–4), displaying clustering stability using 1000 iterations of hierarchical clustering.


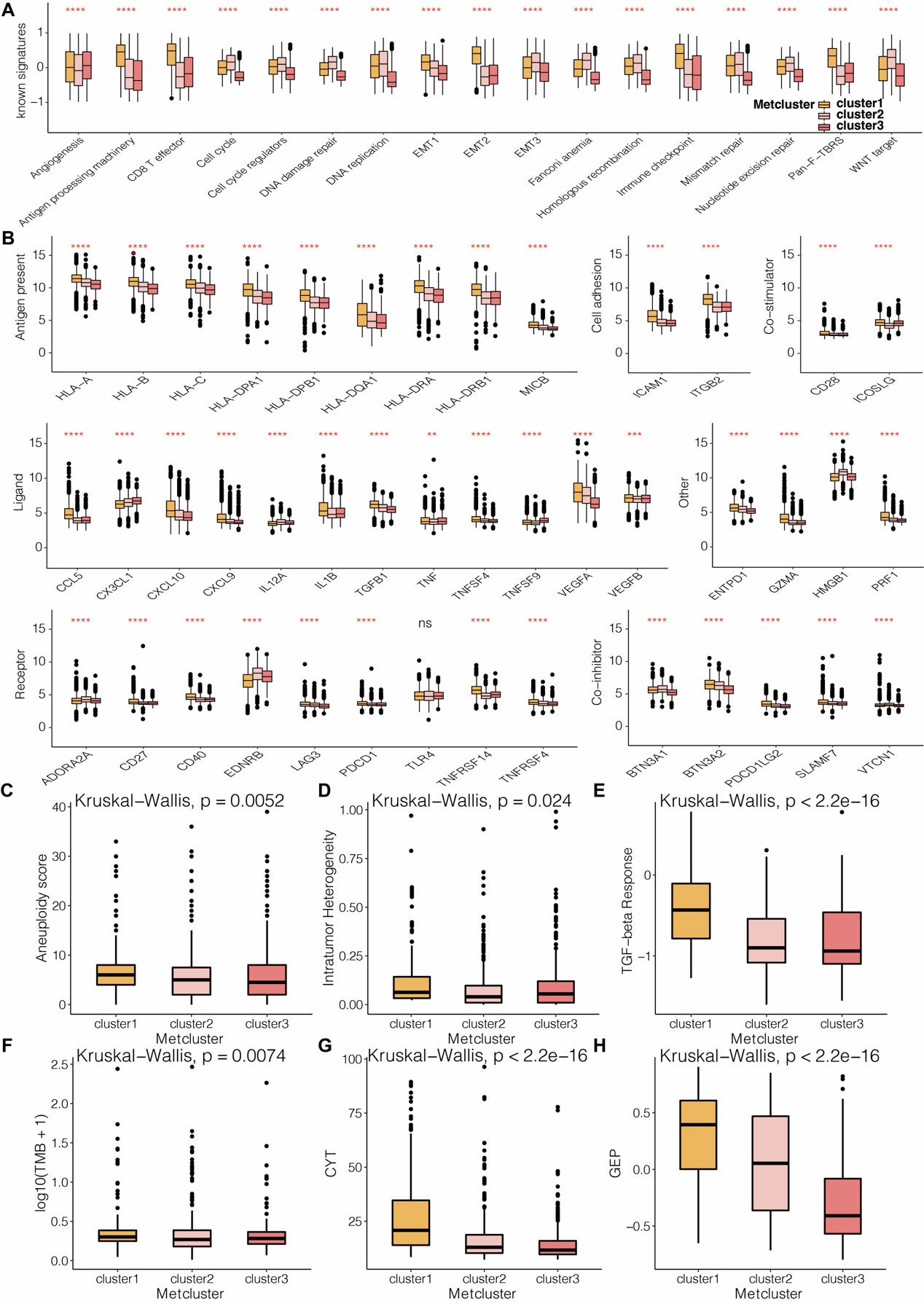


**Figure S2. Immune characteristics of metabolic clusters. A**. Different known signatures distinguished metabolic clusters (immune, mismatch, and stromal signatures as indicated) in the meta-cohort. The scattered dots represent the mean value of signature genes within each group. **B**. Fraction of seven types of immune checkpoints in three metabolic clusters in the meta-cohort. Scattered dots represent immune checkpoint expression values. The thick line represents the median value. The bottom and top of the boxes are the 25th and 75th percentiles (interquartile range). The whiskers encompass 1.5 times the interquartile range. The statistical difference between the three metabolic clusters was compared through the Kruskal–Wallis test. *, P < 0.05; **, P < 0.01; ***, P < 0.001; ****, P < 0.0001. **C**. Aneuploidy score in three metabolic clusters. **D**. Intratumor Heterogeneity in three metabolic clusters. **E**. TGF-β response in three metabolic clusters. **F**. TMB expression difference in three metabolic clusters. **G**. CYT score in three metabolic clusters. **H**. GEP score in three metabolic clusters.


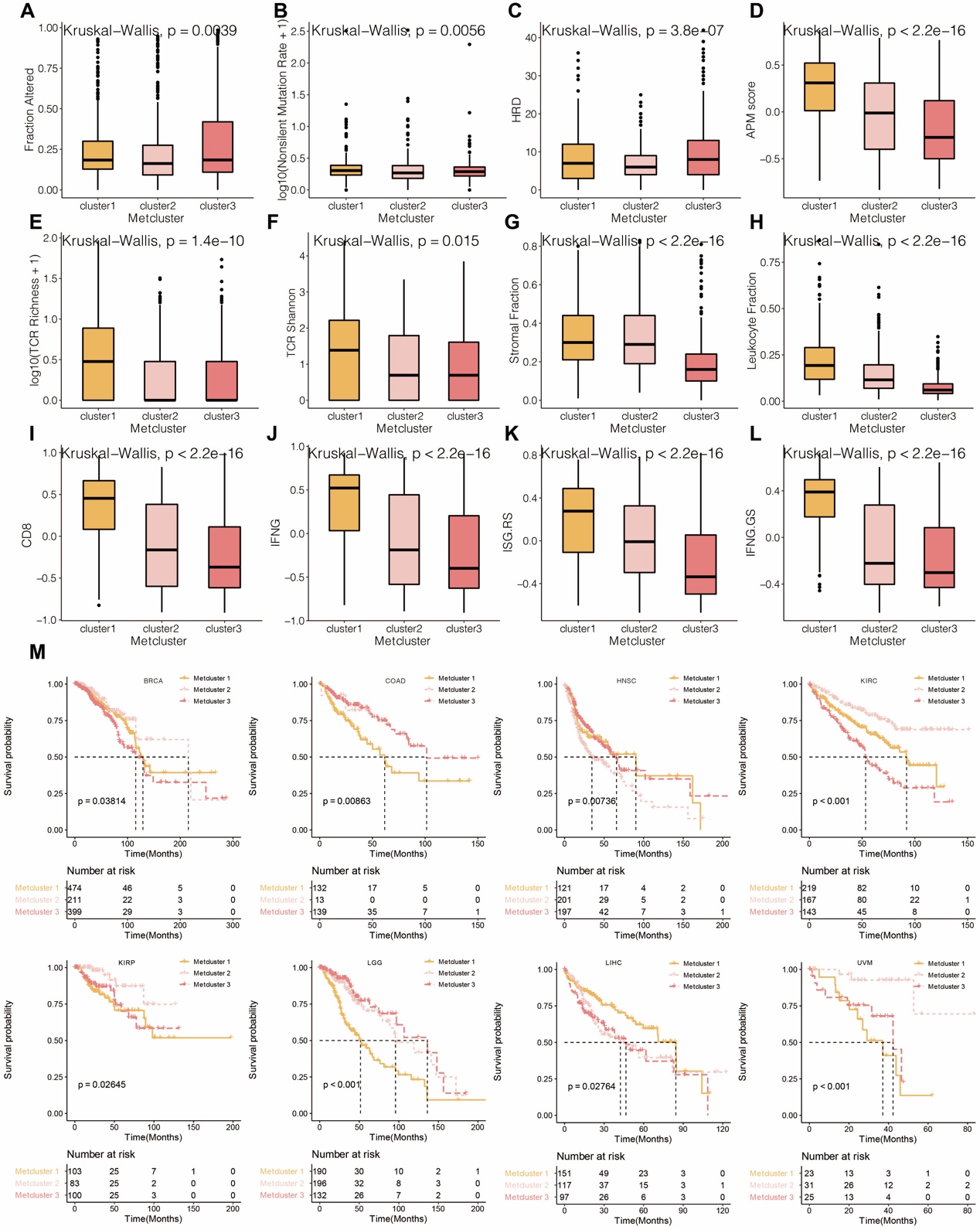


**Figure S3. Immune characteristics and prognostic value of metabolic clusters. A**. Fraction altered in three metabolic clusters. **B**. Nonsilent mutation rate in three metabolic clusters. **C**. HRD in three metabolic clusters. **D**. APM score in three metabolic clusters. **E**. TCR richness in three metabolic clusters. **F**. TCR Shannon in three metabolic clusters. **G**. Stromal fraction in three metabolic clusters. **H**. Leukocyte fraction in three metabolic clusters. **I**. CD8 in three metabolic clusters. **J**. IFNG in three metabolic clusters. **K**. ISG.RS in three metabolic clusters. **L**. IFNG.GS in three metabolic clusters. **M**. Kaplan–Meier curves for three metabolic clusters in pan-cancer datasets, including BRCA, COAD, HNSC, KIRC, KIRP, LGG, LIHC, and UVM.


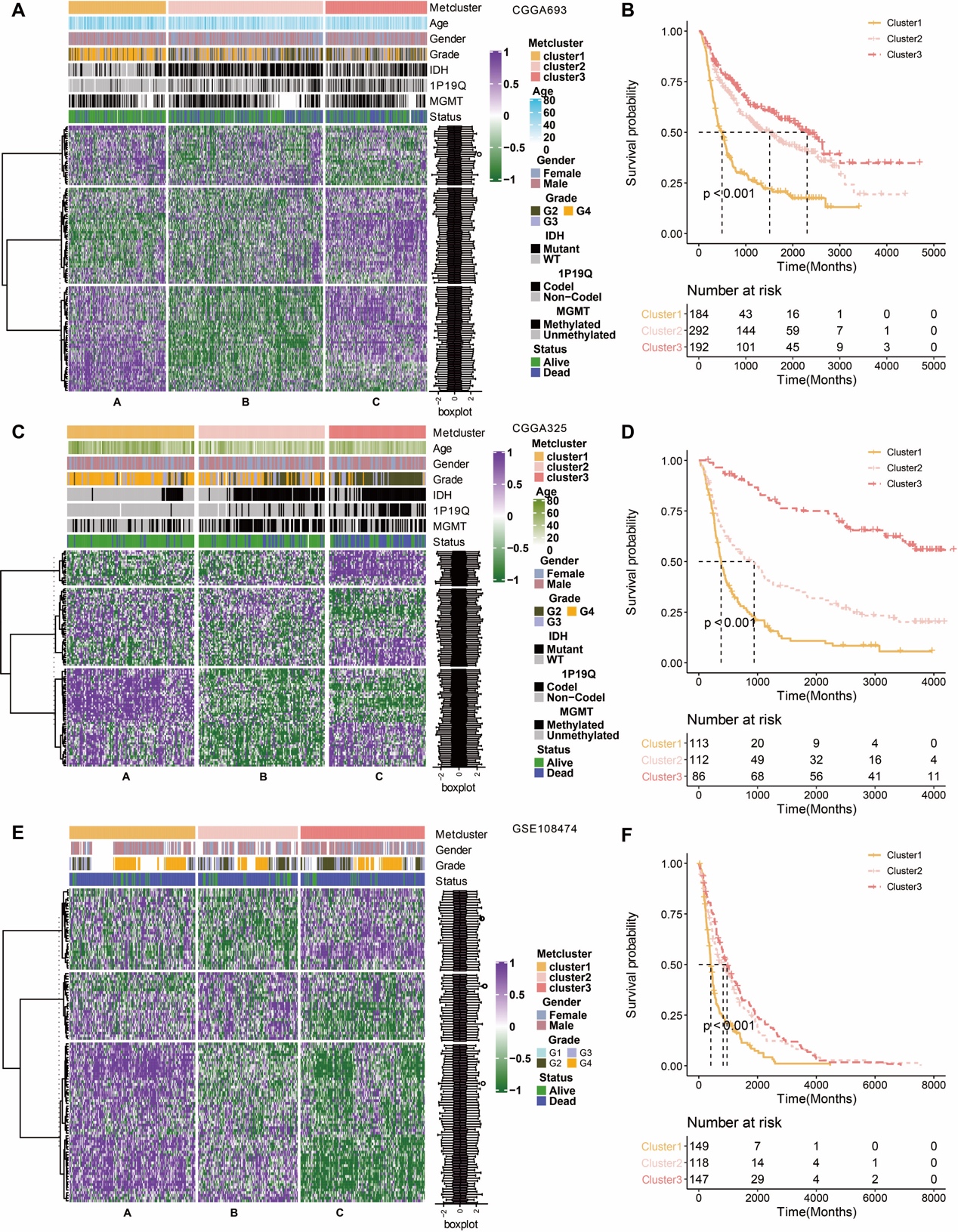


**Figure S4. Construction of three metabolic clusters in external datasets. A.** Unsupervised clustering of metabolism-relevant pathways in CGGA693. **B.** Kaplan–Meier curves for three metabolic clusters in CGGA693. Log-rank test, P < 0.001. **C.** Unsupervised clustering of metabolism-relevant pathways in CGGA325. **D.** Kaplan–Meier curves for three metabolic clusters in CGGA325. Log-rank test, P < 0.001. **E.** Unsupervised clustering of metabolism-relevant pathways in GSE108474. **F.** Kaplan–Meier curves for three metabolic clusters in GSE108474. Log-rank test, P < 0.001.


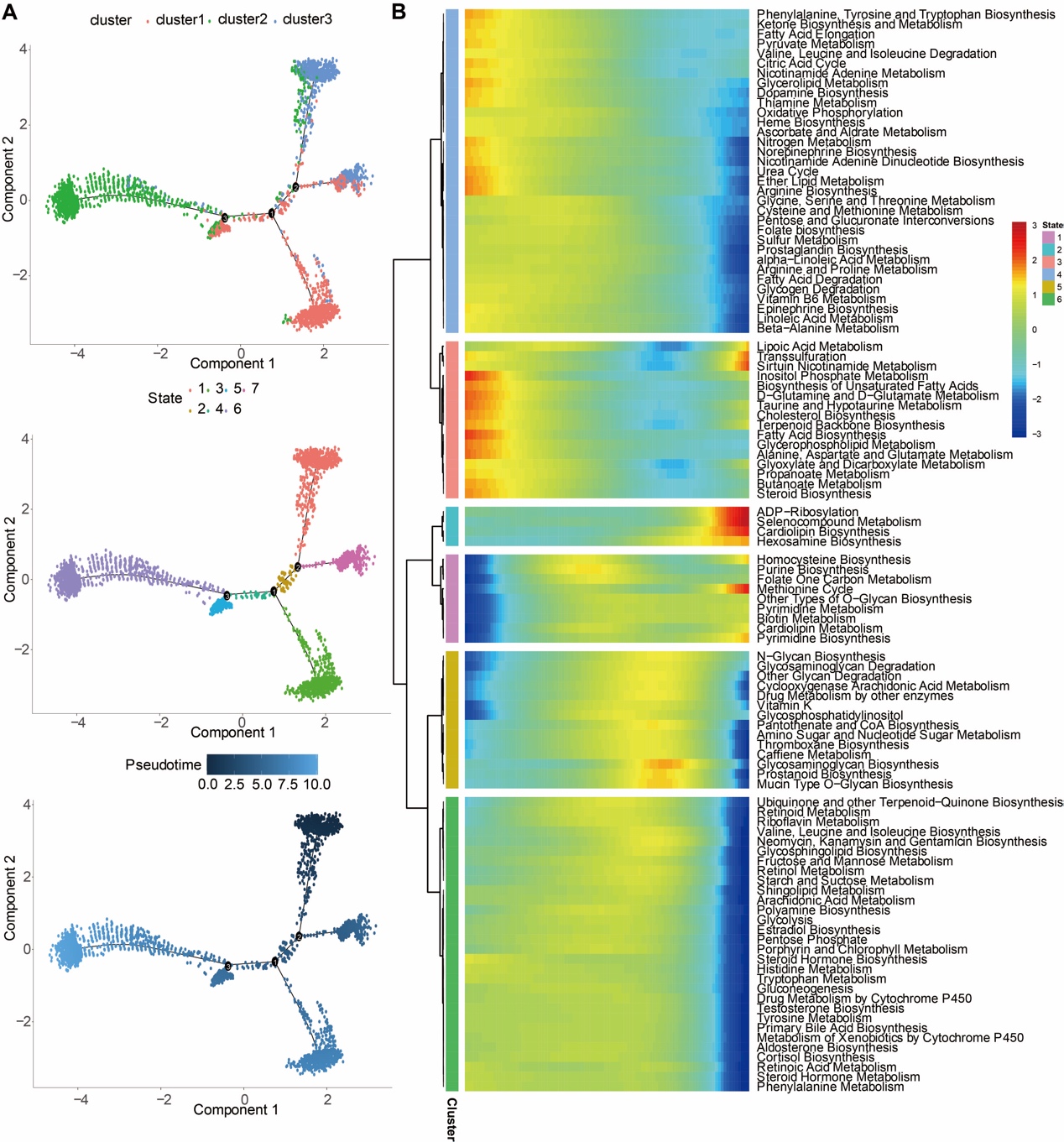


**Figure S5. Reconstruction of a single cell trajectory.** **A**. The single-cell trajectory reconstructed by Monocle contains four main branches. Cells are colored based on the cluster (upper), state (middle), and pseudotime (lower). **B**. Heatmap depicting metabolic signatures with a state-dependent pattern. Each row represents the dynamic expression level of a metabolic-related pathway.


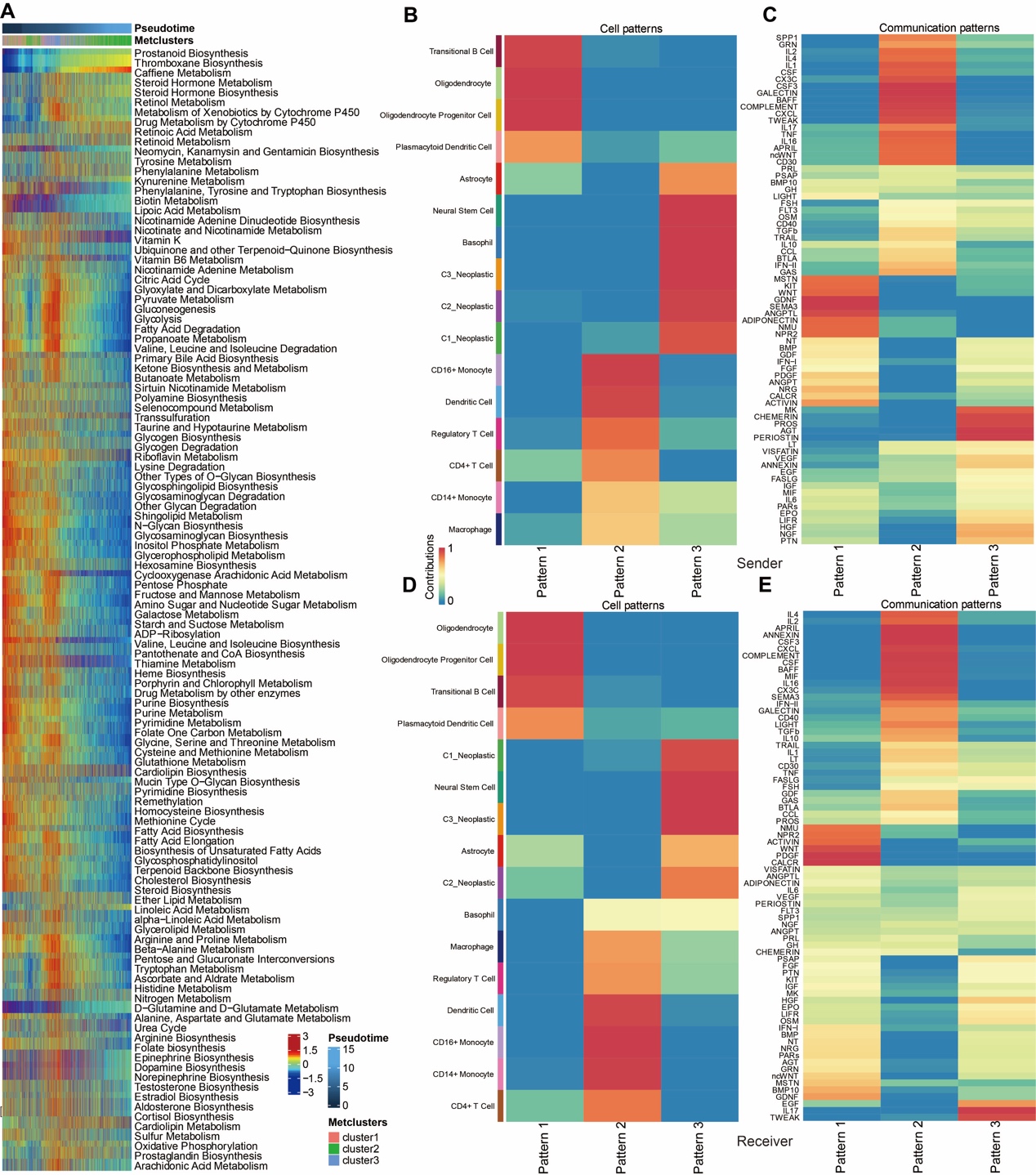


**Figure S6. Functional annotation of metabolic clusters based on single-cell sequencing datasets. A**. Heatmap depicting metabolic signatures with a state-dependent pattern. Each row represents the dynamic expression level of a metabolic-related pathway. **B**. The heatmap of the distribution of 16 cell subtypes across the three sender patterns. **C**. The heatmap for the three communication patterns of the sender is based on the gene expression. **D**. The heatmap of the distribution of 16 cell subtypes across the three-receiver patterns. **E**. The heatmap for the three communication patterns of the receiver is based on the gene expression.


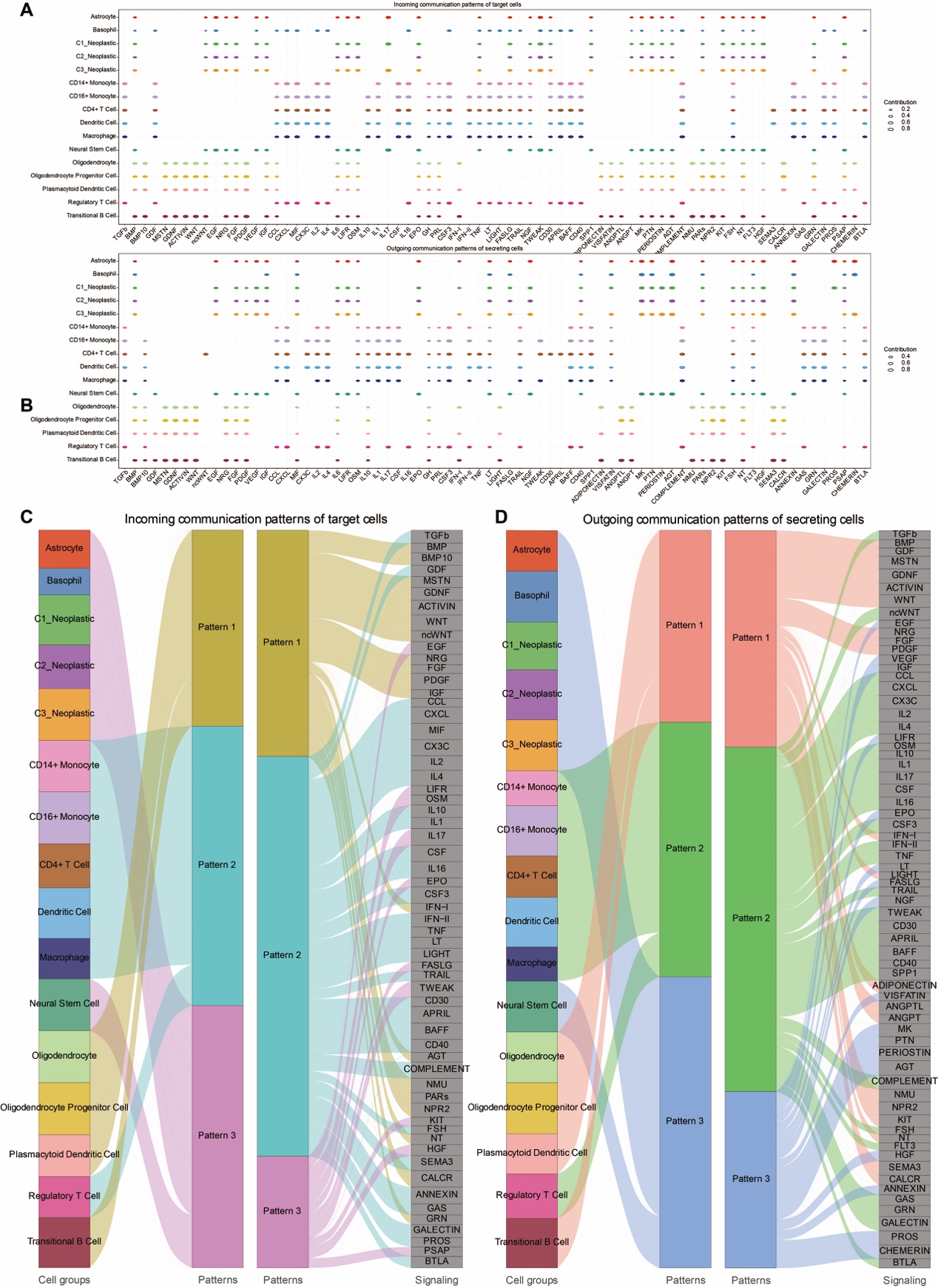


**Figure S7.** **Cellular interaction network. A**. Dot plot depicting the incoming communication pattern of targeted cells. **B**. Dot plot depicting the outcoming communication pattern of targeted cells. **C**. Sankey plot depicting the incoming communication pattern of targeted cells. **D**. Sankey plot depicting the outcoming communication pattern of targeted cells.


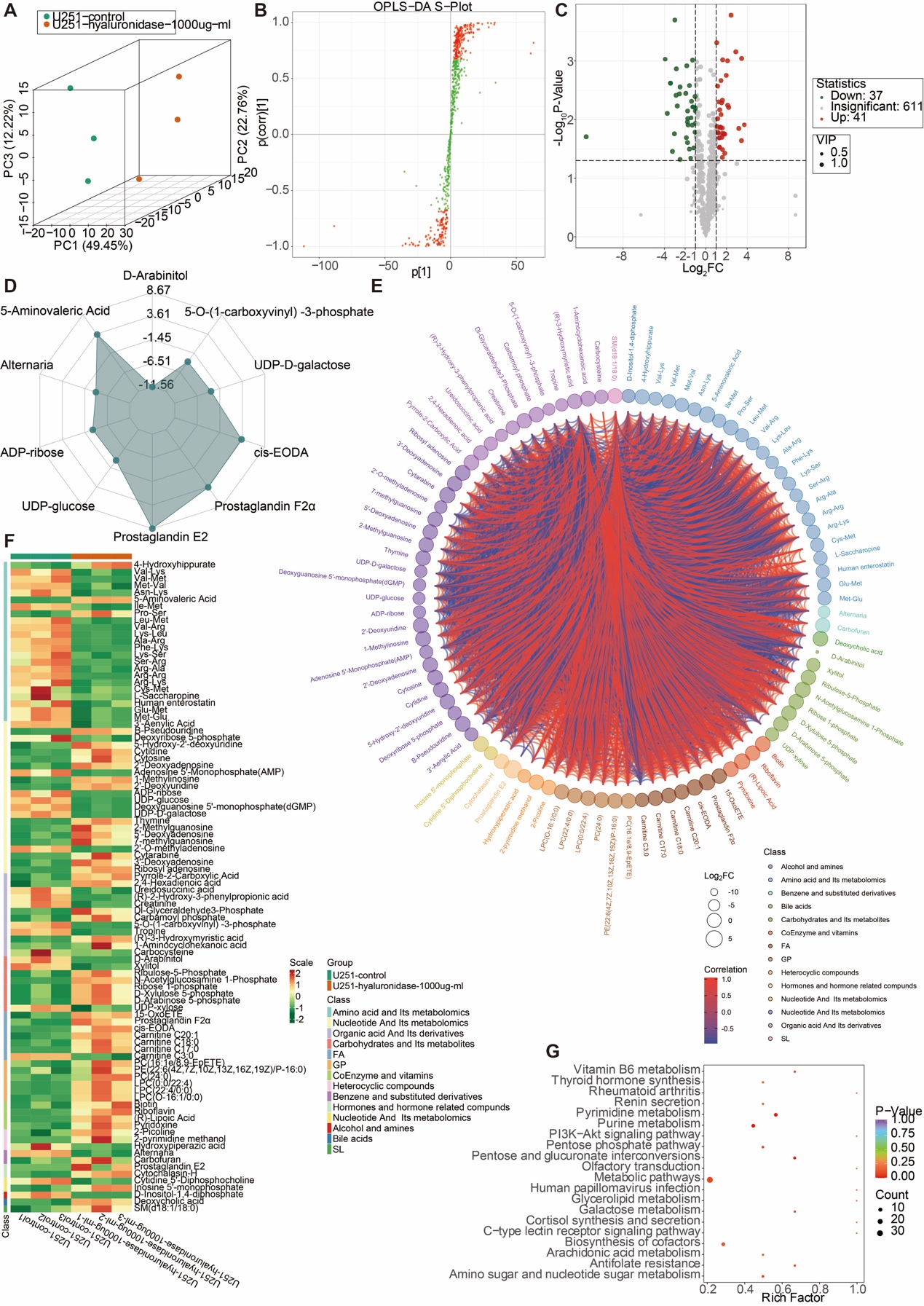


**Figure S8. Metabolomics sequencing in U251 cells. A**. 3D plot for Principal Components Analysis of U251 samples. **B**. S-plot of Orthogonal Projection on Latent Structure-Discriminant Analysis of U251 samples. **C**. Volcano plot of the differentially expressed metabolites between the control group and hyaluronidase group of U251 cells. **D**. Radar plot showing the top 10 differentially expressed metabolites. **E**. Chordal graph showing the interconnection of the differentially expressed metabolites. **F**. Heatmap of the differentially expressed metabolites based on Unit Variance Scaling. **G**. KEGG enrichment analysis of the differentially expressed metabolites. The rich factor is the ratio of the differentially expressed metabolites and the total defined metabolites in the corresponding pathway.


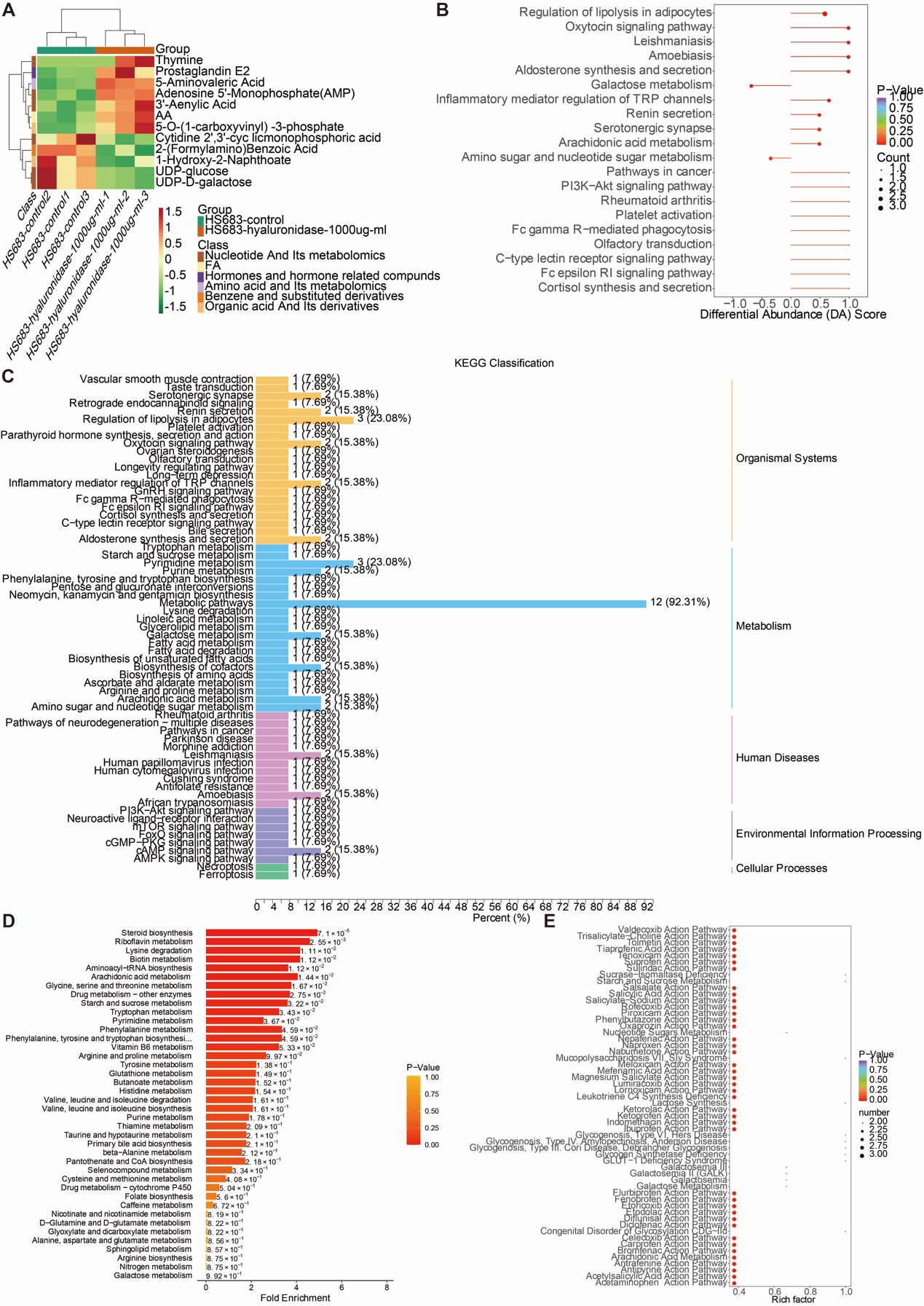


**Figure S9. Functional annotation of the differentially expressed metabolites in HS683 cells. A**. Heatmap of the KEGG pathways with more than five differentially expressed metabolites between the control group and hyaluronidase group of HS683 cells. **B**. The metabolic change of the KEGG pathways is based on the DA score. C. The classification of the identified KEGG pathways. **D**. Metabolite Set Enrichment Analysis based on the differentially expressed metabolites. **E**. The top primary pathways from Human Metabolome Database are based on the differentially expressed metabolites. The rich factor is the ratio of the differentially expressed metabolites and the total defined metabolites in the corresponding pathway.


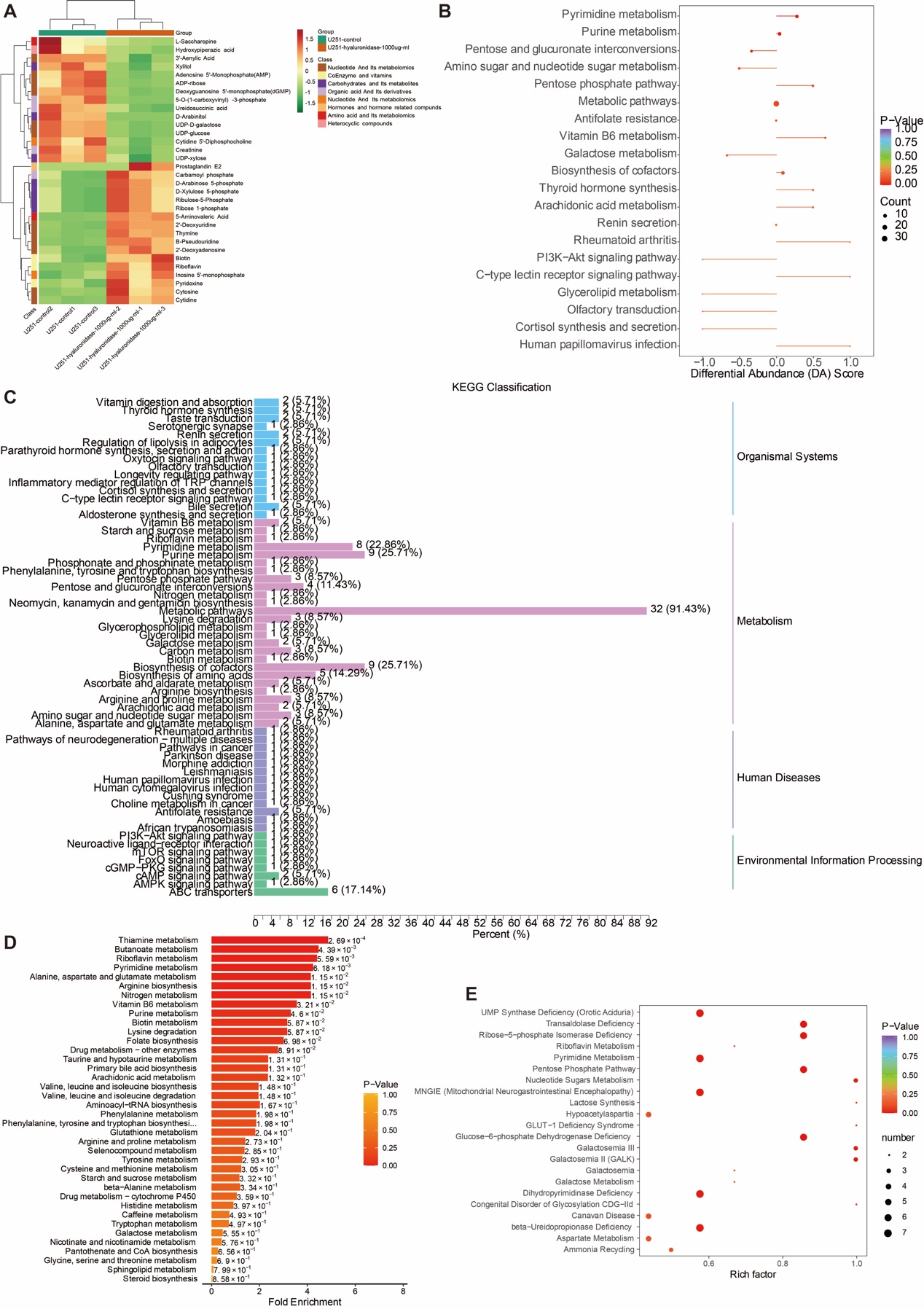


**Figure S10. Functional annotation of the differentially expressed metabolites in U251 cells. A**. Heatmap of the KEGG pathways with more than five differentially expressed metabolites between the control group and hyaluronidase group of U251 cells. **B**. The metabolic change of the KEGG pathways is based on the DA score. C. The classification of the identified KEGG pathways. **D**. Metabolite Set Enrichment Analysis based on the differentially expressed metabolites. **E**. The top primary pathways from Human Metabolome Database are based on the differentially expressed metabolites. The rich factor is the ratio of the differentially expressed metabolites and the total defined metabolites in the corresponding pathway.
